# An Out-of-Patagonia migration explains the worldwide diversity and distribution of *Saccharomyces eubayanus* lineages

**DOI:** 10.1371/journal.pgen.1008777

**Published:** 2020-05-01

**Authors:** Roberto F. Nespolo, Carlos A. Villarroel, Christian I. Oporto, Sebastián M. Tapia, Franco Vega-Macaya, Kamila Urbina, Matteo De Chiara, Simone Mozzachiodi, Ekaterina Mikhalev, Dawn Thompson, Luis F. Larrondo, Pablo Saenz-Agudelo, Gianni Liti, Francisco A. Cubillos

**Affiliations:** 1 Instituto de Ciencias Ambientales y Evolutivas, Universidad Austral de Chile, Valdivia, Chile; 2 Millennium Institute for Integrative Biology (iBio), Santiago, Chile; 3 Center of Applied Ecology and Sustainability (CAPES), Santiago, Chile; 4 Universidad de Santiago de Chile, Facultad de Química y Biología, Departamento de Biología, Santiago, Chile; 5 Université Côte d’Azur, CNRS, INSERM, IRCAN, Nice, France; 6 Ginkgo Bioworks, Boston, Massachusetts, United States of America; 7 Departamento Genética Molecular y Microbiología, Facultad de Ciencias Biológicas, Pontificia Universidad Católica de Chile, Santiago, Chile; University of Laval, CANADA

## Abstract

Population‐level sampling and whole‐genome sequences of different individuals allow one to identify signatures of hybridization, gene flow and potential molecular mechanisms of environmental responses. Here, we report the isolation of 160 *Saccharomyces eubayanus* strains, the cryotolerant ancestor of lager yeast, from ten sampling sites in Patagonia along 2,000 km of *Nothofagus* forests. Frequency of *S*. *eubayanus* isolates was higher towards southern and colder regions, demonstrating the cryotolerant nature of the species. We sequenced the genome of 82 strains and, together with 23 available genomes, performed a comprehensive phylogenetic analysis. Our results revealed the presence of five different lineages together with dozens of admixed strains. Various analytical methods reveal evidence of gene flow and historical admixture between lineages from Patagonia and Holarctic regions, suggesting the co-occurrence of these ancestral populations. Analysis of the genetic contribution to the admixed genomes revealed a Patagonian genetic origin of the admixed strains, even for those located in the North Hemisphere. Overall, the Patagonian lineages, particularly the southern populations, showed a greater global genetic diversity compared to Holarctic and Chinese lineages, in agreement with a higher abundance in Patagonia. Thus, our results are consistent with a likely colonization of the species from peripheral glacial refugia from South Patagonia. Furthermore, fermentative capacity and maltose consumption resulted negatively correlated with latitude, indicating better fermentative performance in northern populations. Our genome analysis, together with previous reports in the sister species *S*. *uvarum* suggests that a *S*. *eubayanus* ancestor was adapted to the harsh environmental conditions of Patagonia, a region that provides the ecological conditions for the diversification of these ancestral lineages.

## Author summary

Lager yeast history has intrigued scientists for decades. The recent isolation of *S*. *eubayanus*, the lager yeast ancestor, represents an unprecedented opportunity to extend our knowledge on yeast phylogeography and the origins of the *S*. *pastorianus* lager hybrid. However, the genetic, phenotypic and evolutionary history of this species remains poorly known. Our work demonstrates that *S*. *eubayanus* isolates from Patagonia have the greatest genetic diversity, comprising the largest number of lineages within a single geographic region and experienced ancestral and recent admixture between lineages, likely suggesting co-occurrence in Patagonia. Importantly, some isolates exhibited significant phenotypic differences for traits such as high temperature and ethanol tolerance, together with fermentation performance, demonstrating their potential in the brewing industry for the generation of new styles of lager beers. Furthermore, our results support the idea of colonization from peripheral glacial refugia from the South, as responsible for the high genetic diversity observed in southern Chilean Patagonia. Our results allow hypothesizing a successful physiological adjustment of the species to the local conditions in Patagonia, explaining its wide distribution in the southern hemisphere.

## Introduction

The identification of admixed and/or hybrid individuals in nature together with the geographic distribution of ecologically relevant traits is crucial for the understanding of how new lineages diversify in a given climate/region/environment [1]. Unfortunately, studies addressing admixture in fungi are still uncommon. In this context, the utilization of population‐level sampling and whole‐genome sequences involving large sets of individuals allows studying genetic correlations by exploring patterns of genomic variation across lineages and identifying signatures of gene flow, revealing potential mechanisms of physiological adjustment to the environment [2]. The monophyletic *Saccharomyces* genus is an ideal model to investigate genomic variation and admixture, since within the clade isolates occupy a broad range of environments. Currently, the clade is composed of eight species [3], including the partially domesticated *S*. *cerevisiae* and other non-domesticated species, such as *S*. *eubayanus* [4]. Given the economic importance of this clade, as well as the wealth of genomic information that has been produced in the past decade, particularly for the model organism *S*. *cerevisiae* [5–10], natural populations of *Saccharomyces* are excellent models for understanding genome evolution in the wild.

Having been the first sequenced eukaryote, studies in *S*. *cerevisiae* have gained great depth by the wealth of additional full genome data coming from diverse isolates, providing exceptional new insights into the genomic processes that drive phenotypic traits and genome evolution between isolates [5, 6, 11]. Since the recent possibility to rapidly and cost-effectively sequence full genomes, other *Saccharomyces* genomes have been fully obtained [3]. *Saccharomyces* species harbour different genetic distributions, population histories and unique phenotypic properties [12]. Phylogenomics analyses in *S*. *uvarum* suggested that a Patagonian sub-population gave rise to the Holarctic population through a recent bottleneck [13]. Similarly, recurrent hybridization events between lineages were reported in *S*. *paradoxus*, demonstrating how recurring hybridization events in nature contribute to genomic, phenotypic and potentially the species diversity [14]. In this context, admixture in yeast has been associated to transient habitats, like fruits, where insects can carry live yeast spores and favour a wider geographic distribution and genetic admixture compared to less nutrient rich barks, where admixture could occur over longer periods [15, 16].

In nature, several *Saccharomyces* inter-species hybrids have been found. An example of this includes the workhorse of the modern brewing industry, *S*. *pastorianus*, a hybrid between *S*. *cerevisiae* and the cold-tolerant *S*. *eubayanus* [17, 18]. Despite the industrial importance of *S*. *pastorianus*, much of the natural history of this hybrid remains obscure, largely because the *S*. *eubayanus* parental species was only recently isolated [19]. *S*. *eubayanus* was originally isolated from *Nothofagus* trees in the Argentinian Patagonia [19, 20] and since then it has been isolated in New Zealand [21], North America [22, 23] and East Asia [24]. However, the evolutionary origin of *S*. *eubayanus* is still controversial. While this species has been isolated from South American *Nothofagus* trees recurrently [20] and only a handful of isolates have been recovered from trees in China and North America [24, 25], a subset of the strains from China have been reported as the earliest diverging lineage, suggesting an Asian origin of the species [24]. However, these findings have been challenged [20]. Molecular profiling indicates that *S*. *eubayanus* is composed of three lineages, besides the early diverging lineage of West China. These populations include a ‘Holarctic’ (HOL) cluster (a group of related strains from Tibet and North America) and two Patagonian populations denominated: ‘Patagonia A’ (PA) and ‘Patagonia B’ (PB) [25]. Whole genome sequence comparison among wild *S*. *eubayanus* strains indicates that, thus far, the Holarctic lineage is the closest relative of the lager yeast [20, 25]. Interestingly, multi-locus sequence comparisons have indicated that the nucleotide diversity of *S*. *eubayanus* Patagonian populations is higher than that of the West China and the Holarctic (North American) lineages. However, until now only a handful of *S*. *eubayanus* genomes per population have been fully sequenced, which prevents depicting a detailed population genomics portrait of this species.

**Fig 1:**
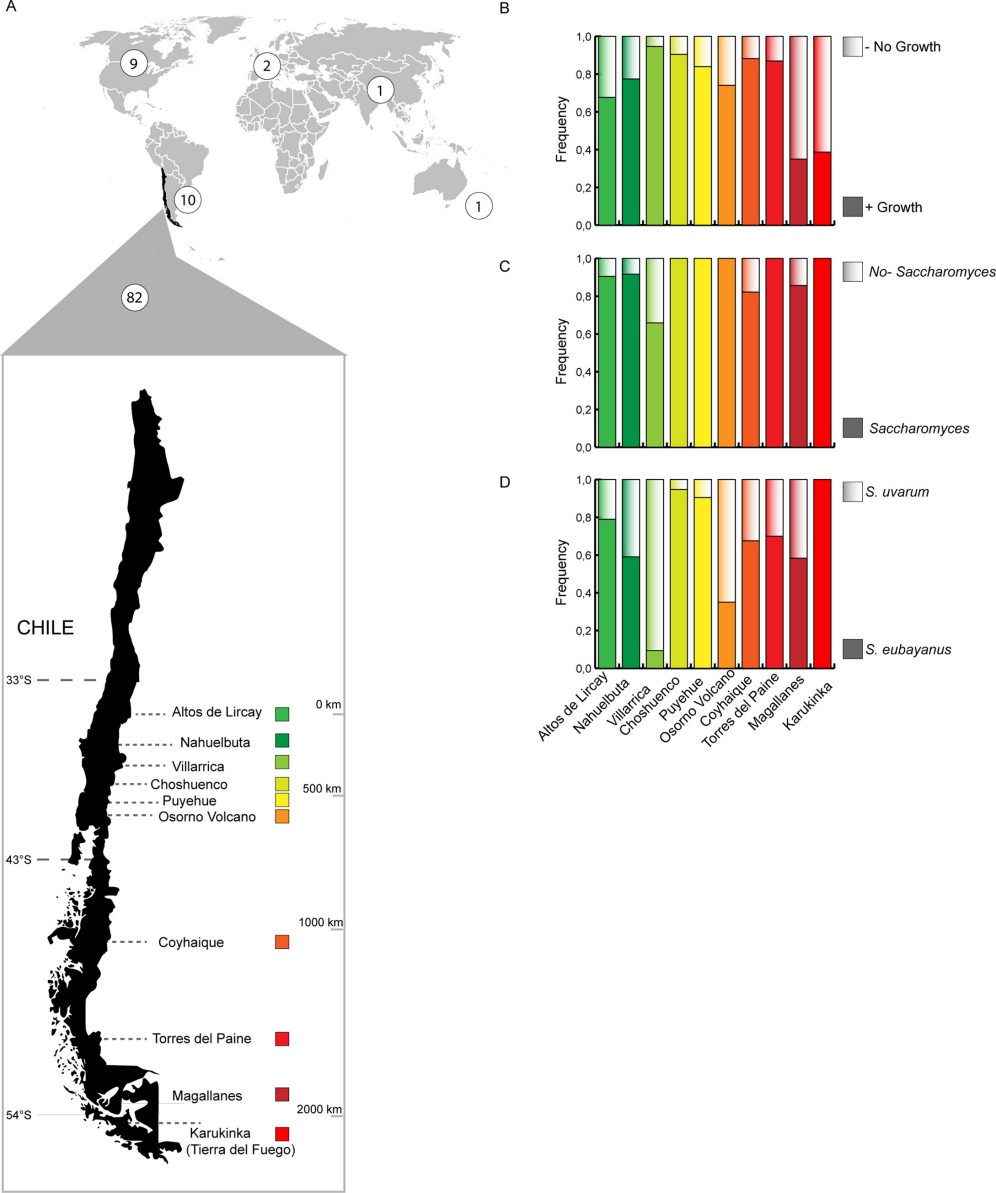
Geographic distribution and isolation frequency of *S*. *eubayanus* strains. (A) Map of the world depicting the number of available *S*. *eubayanus* sequenced genomes from around the world (white circles), together with the ten localities in Chile where the 82 strains sequenced in this study were isolated. Overall, a latitude range of 2,090 km was covered. Frequency of bark samples that yielded a (B) successful yeast isolation (full coloured) or no growth in YNB raffinose ethanol media (shaded colours), a (C) *Saccharomyces* (full coloured) or other non-*Saccharomyces* genera (shaded colours), and a (D) *S*. *eubayanus* (full coloured) or *S*. *uvarum* (shaded colours) species.

Here, we describe the isolation of 160 *S*. *eubayanus* strains from bark samples obtained from *Nothofagus* trees in Chile (**Fig 1**) and we provide annotated genomes together with phenotypic characterization for 82 selected strains. We investigated genetic structure, admixture and nucleotide diversity of this set of strains, while also re-analyzed 23 previously published genomes. Overall, we provide evidence of historical admixture events in Patagonia that significantly expands the formerly known genetic history of the species. Moreover, phenotypic clustering correlated well with genetic distances, where individuals from northern sites showed greater fermentation performance and high-temperature tolerance than isolates from southern sites. The genomic data presented here broadens our knowledge of the genetics, ecology, and evolution of wild yeast strains.

## Results

### *S*. *eubayanus* isolation and whole genome sequencing

In order to determine the distribution of *S*. *eubayanus* along the south western side of the Andes Mountains, we sampled ten Chilean national parks and reserves between 2017 and 2018, spanning 2,090 km from Altos de Lircay National Park in central Chile (VII Maule Region, Chile) to Karukinka Natural Park in southern Chile (XII Magallanes Region, Chile) (**Fig 1A**). From these sites, we obtained 553 bark samples from trees belonging to *N*. *pumilio*, *N*. *Antarctica*, *N*. *dombeyi* and *A*. *Araucana* species. Raffinose and ethanol media enrichment [26] allowed us to recover yeast colonies in 77% of the samples. Potential *Saccharomyces* strains were identified by sequencing the ITS and/or *GSY1* and *RPI1* RFLP [22, 25]. From these, 160 *S*. *eubayanus* strains were identified from different individual trees (**S1 Table,** representing 28.9% of the samples), and in parallel, another set of 179 *S*. *uvarum* isolates were recovered (representing 37.9% of the samples), together with dozens of non-*Saccharomyces* species belonging to the *Lachancea*, *Kregervanrija*, *Kazachstania* and *Hanseniaspora* genera. In general, we observed a pattern between yeasts and hosts, where *N*. *pumilio* and *N*. *antarctica* contained mostly *S*. *eubayanus* strains, while all but one isolate derived from *N*. *dombeyi* and *A*. *araucana* samples were identified as *S*. *uvarum* (**S1 Table**). The frequency of yeast isolates was higher towards southern regions, while a lower fraction of yeast colonies were obtained from Tierra del Fuego. FACS analysis revealed that all samples were diploids, except for CL609.1 and CL1005.1 which were found to be haploid and tetraploid, respectively (**S1 Fig**). Indeed, all strains were able to sporulate, with the exception of CL609.1. Overall, our results demonstrate the high frequency of the *S*. *eubayanus* species above latitude 33° in the western side of the Andes Mountains.

### Population structure in *S*. *eubayanus*

**Fig 2:**
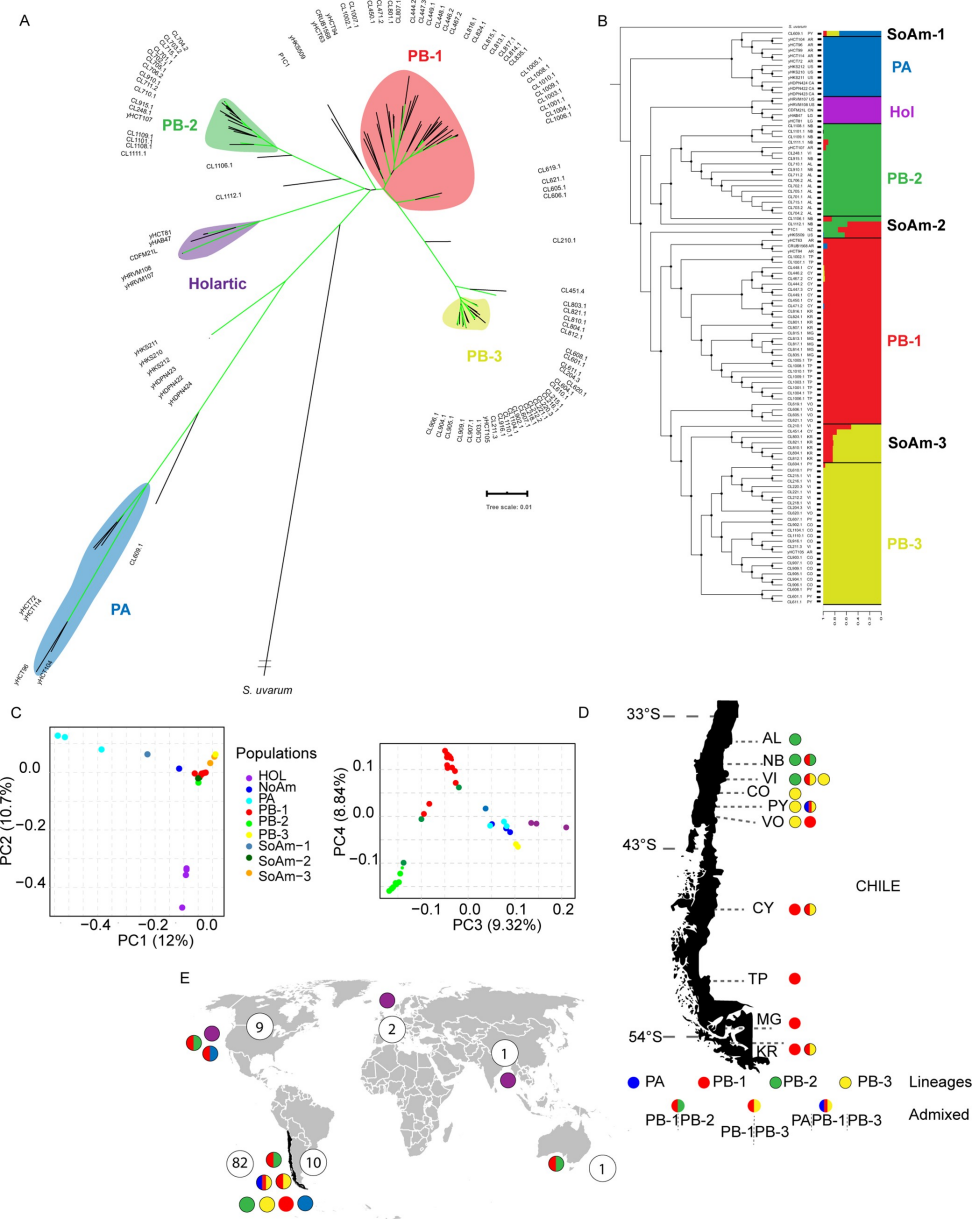
Phylogeny of *S*. *eubayanus*. (A) Maximum likelihood tree depicting genetic relationships between 104 strains using 606,656 biallelic SNPs (substitution model GTR+F+ASC) and manually rooted with *S*. *uvarum* as the outgroup. Green coloured branches indicate bootstrap support values greater than 90%. Three PB lineages: PB-1 (red), PB-2 (green) and PB-3(yellow) and a single PA lineage (blue) were identified, together with admixed strains between the different lineages. Branch lengths correspond to genetic distance. Tree scale is substitutions per site. (B) Maximum likelihood tree of 104 strains together with the Bayesian clustering output generated with STRUCTURE. An optimum k = 5 groups is shown. The geographic origin of each strain is depicted as follows: Canada (CA), United States (UN), China (CN), Lager (LG), AR (Argentina), New Zeland (NZ), AL (Altos de Lircay), NB (Nahuelbuta), Villarrica (VI), Choshuenco (CO), Puyehue (PY), Osorno Volcano (VO), Coyhaique (CY), Torres del Paine (TP), Magallanes (MG) and Karukinka (KR).(C) Plot of the distribution of the genomic variation in 83 non-admixed strains based on the first two components, third and fourth of a PCA analysis performed using 80,203 unlinked SNPs. Colour codes used are congruent with clusters presented in panel A. Each dot represents a single strain. Distribution across Chile (D) and the rest of the world (E) for Patagonian lineages and admixed lineages. Colour codes correspond to different genetic lineages obtained with Structure. Coloured circles in (D) and (E) are representative of pure lineages (single colour) and admixed strains (two or three coloured-circles). Numbers in circles correspond to the number of sequenced strains. In (E) Patagonian lineages from Chile and Argentina are included.

To investigate the genomic variation and population structure of the *S*. *eubayanus* isolates, we sequenced the genomes of 82 strains, randomly selected from the ten sampling sites. Furthermore, we combined our dataset with all previously published genome from 23 strains from North America (9 strains), Argentina (10 strains) [25], China (1 strain) [24], New Zealand (1 strain) [21] and two lager genomes [18], allowing us to incorporate genomes obtained in other geographical regions (**S2A Table and S2B Table**). Overall, no strain isolated in this study represents a close relative to the type strain (obtained from Argentina), suggesting that the Andes Mountains represents a natural barrier between *S*. *eubayanus* populations (**S2B Table**). On average, across the 82 genomes we obtained 39,024 SNPs per strain relative to the reference genome, and a SNP was found on average every ~300 bp. In parallel, we found on average 1,606 insertions and 1,677 deletions per isolate relative to the type strain (**S2A Table**). The phylogeny obtained suggests that Chilean strains displayed different ancestry and mostly fell into three major lineages: PB-1, PB-2 & PB-3 (**Fig 2A**). Furthermore, several strains fell outside the major PB lineages and might represent admixed strains (**Fig 2A**).

In order to study the genetic structure of *S*. *eubayanus*, we used STRUCTURE, fineSTRUCTURE clustering and principal component analysis (PCA). All analyses revealed a high degree of differentiation across the populations (**Fig 2)**. STRUCTURE analysis indicated an optimum k = 5 groups (ΔK_5_ = 2,652, **Fig 2B, S3 Table**), whereas fineSTRUCTURE was able to depict several subpopulations within the PB-1 lineage, and to a lower extent also in PB-3 and PB-2, respectively (**S2 Fig**), in agreement with our PCA analysis (**Fig 2C**). Moreover, three groups of strains showed a mixture of alleles inherited from multiple populations (SoAm 1–3), that together with the North American (NoAm), Holarctic and PA lineages shape the genetic structure of S. *eubayanus* (**Fig 2D and 2E**).

**Fig 3:**
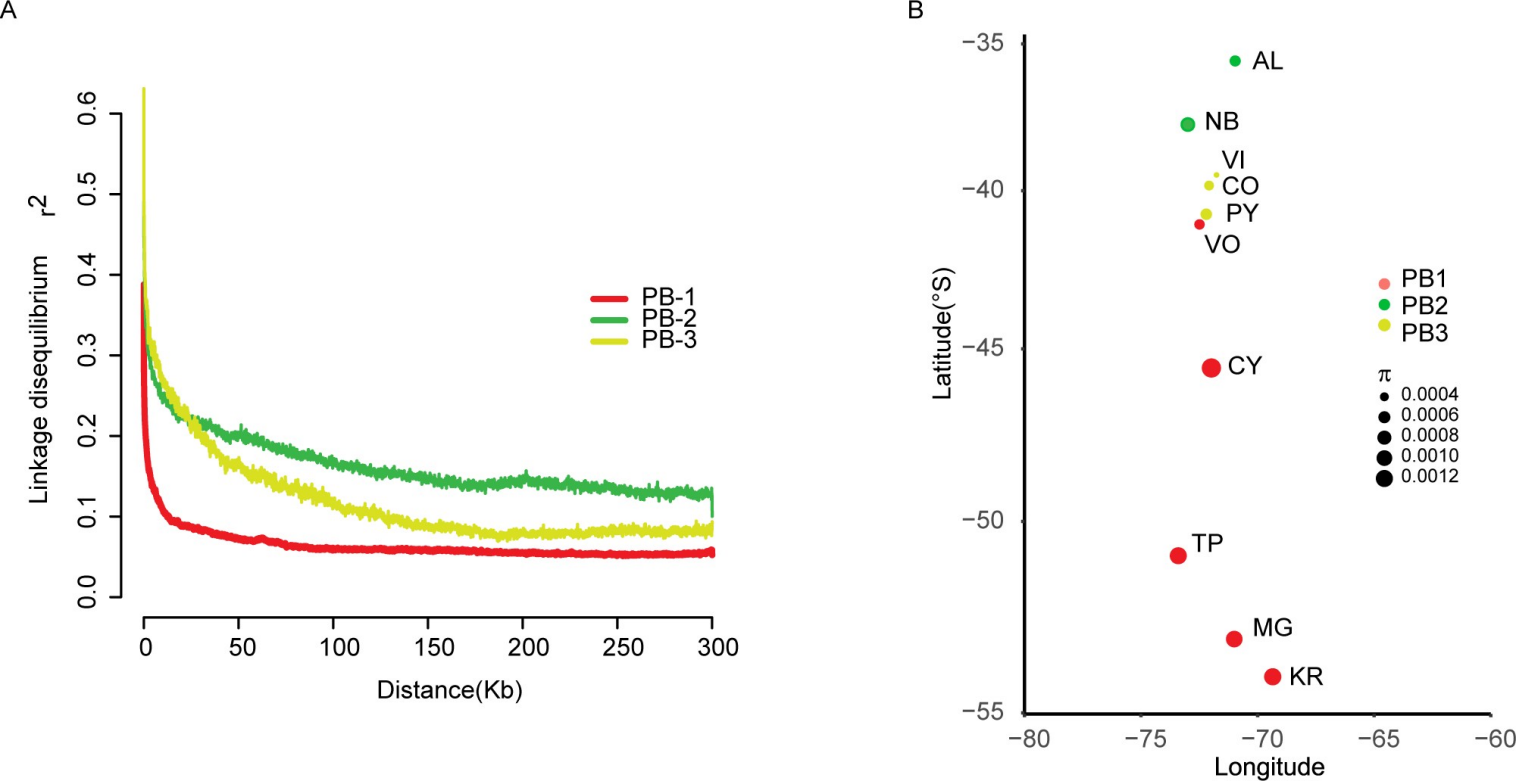
Linkage disequilibrium and genetic diversity across lineages. (A) Linkage disequilibrium decay over distance (kb) expressed in terms of correlation coefficient, r2. LD decay for each window was estimated as the pairwise average for all SNPs pairs separated by no more than 100 kb. The PB-1 lineage shows the lowest LD values compared to any other population in our collection. (B) Nucleotide diversity (π) in localities across Chile. Colours represent the different PB lineages as indicated.

In order to detect evidence of recombination in the different lineages, we estimated linkage disequilibrium (LD) using only non-admixed strains. Estimates of LD based on *r*^*2*^ values differed between the three PB lineages (**Fig 3A**). Lineages showed a 50% LD decay of 2.9 kb, 29.1 kb and 22.5 kb in the PB-1, PB-2 and PB-3 populations, respectively, demonstrating a population-specific LD decay and greater LD levels in the PB-1 population. Moreover, the PB-1 level of recombination was similar to what is described in domesticated *S*. *cerevisiae* populations [5, 7]. This could be explained by greater inbreeding or outbreeding rates in the lineage. Indeed, *F*is values (Wright’s inbreeding coefficient) were significantly higher (*p*-value < 0.001, paired Student t-test) in PB-1 (average *F*is = 0.9482 CI = 0.9462–0.9503), compared to PB-2 and PB-3 (average *F*is = 0.9017 (CI = 0.8974–0.906 & *F*is = 0.8856 CI = 0.8795–0.8916, respectively) (**S4A Table**). Alternatively, lower LD levels for PB-2 and PB-3, could be due to the hidden population substructure within these two lineages compared to PB-1, impacting the overall comparison.

Next, we calculated nucleotide diversity (π), genetic differentiation (*F*_*ST*_), and neutrality test statistics, such as Tajima’s D (**S4A Table**). In general, we observed a trend for higher genome-wide nucleotide diversity (π) in southern localities, and belonging to PB-1, compared to their northern counterparts belonging to PB-2 and PB-3 (**Fig 3B**). In this context, PB-3 isolates from Choshuenco and Villarrica, located further north had the lowest levels of genetic diversity. These results demonstrate a negative and significant (*p*-value = 0.047, Spearman correlation coefficient rs = 0.632) correlation between latitude and genetic diversity.

Tajima’s D scores differed between lineages. Specifically, Tajima’s D values for PB-2 & PB-3 were positive while this metric was near zero for PB-1, suggesting undetected population substructure in the former case and no population decline for the latter (**S4A Table**, **S3A Fig**). We then assessed the degree of genetic differentiation and nucleotide diversity between the ten sampling sites per lineage (**S3B Fig**). We found moderate to high significant *F*_*ST*_ values ranging from 0.16 to 0.88 (**S4B Table**). A mantel test showed a significant correlation between the geographic and the genetic distances (isolation by distance, *p*-value < 0.05) among sampling sites of the PB-1 lineage. The number of pairwise comparisons was insufficient to test for IBD for lineages PB-2 and PB-3. The positive IBD for PB-1 indicates limited effective dispersal within this lineage (**S3C Fig**).

### Historical recombination events suggest a Patagonian genetic origin of admixed strains

**Fig 4:**
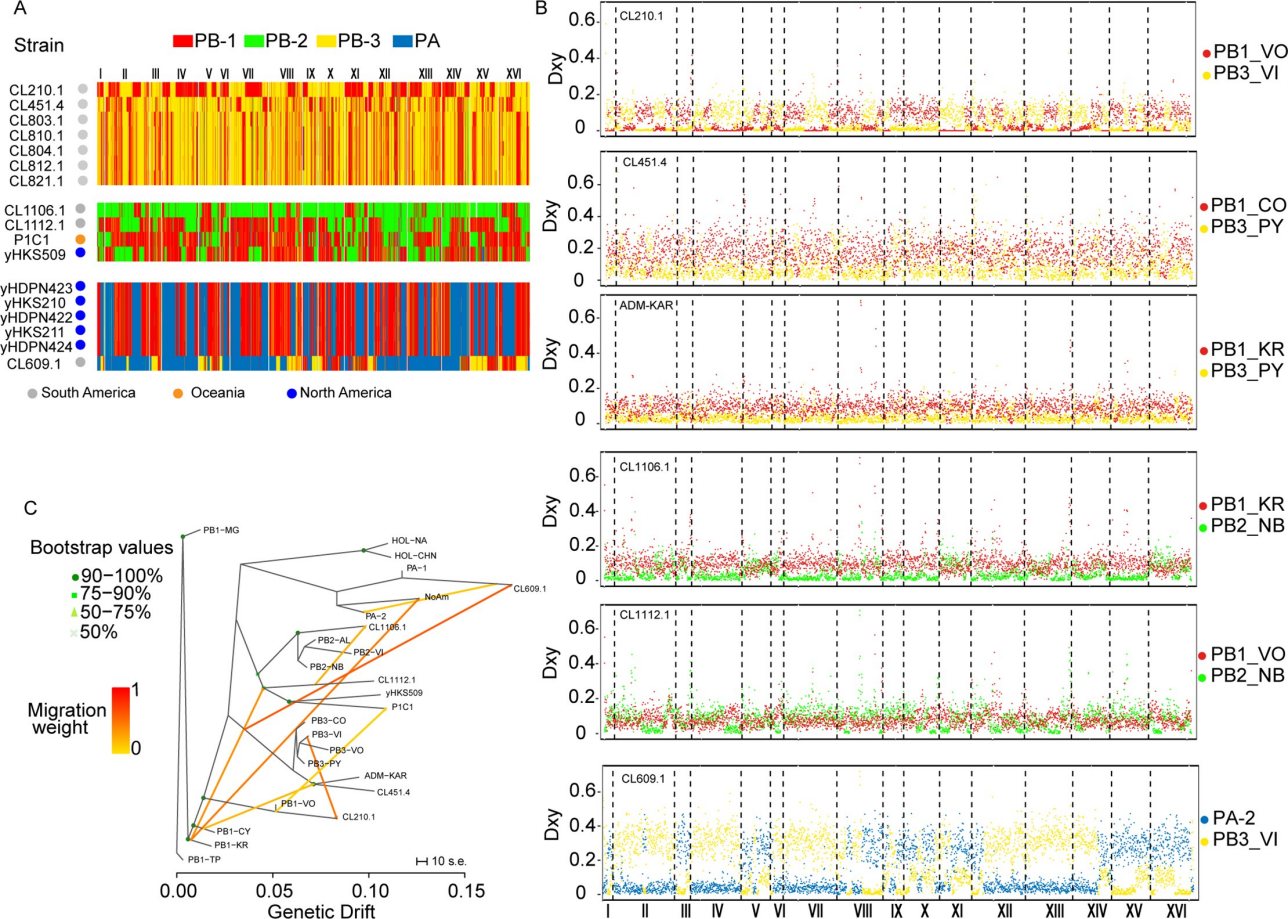
Parental lineages in admixed genomes. (A) Genome-wide ancestry for admixed strains. Bins of 100 SNPs were assigned in the admixed strains to the populations PB-1 (red), PB-2 (green), PB-3 (yellow) or PA (blue) based on sequence similarity. Chromosomes are indicated in the Y axis from I to XVI, while in the X-axis strains are depicted. Two colours in the same row (strain) suggest admixed genomes. (B) Pairwise divergence of Karukinka admixed (ADM-KAR) population and other Chilean admixed strains from parental lineages. Red, green, yellow, and light blue dots indicate sequence divergence of the admixed individual/population to their putative parental subpopulations predicted by GLOBETROTTER. For this analysis admixed genomes were divided into bins of 100 SNPs (C) Phylogenetic network inferred by Treemix in the admixed isolates evidencing recent hybridization events. Eight migration edges between populations were allowed and are shown with arrows indicating the direction toward the recipient group and coloured according to the ancestry percentage received from the donor. The scale bar shows ten times the average standard error of the entries in the sample covariance matrix.

To determine how recombination events influenced the genomes of the isolates showing recent genetic admixture and the level of genetic exchange between populations, we explored their genome compositions and genetic origins. First, we generated similarity plots using 100 SNPs blocks and determined the closest genetic origin for each of the three groups of admixed strains from each population. This initial assessment of admixture allowed us to determine that strains isolated from Karukinka (SoAm-3) likely originated from the same admixture event between PB-1 and PB-3 lineages, as 92% of their bins were assigned to the same lineage (**Fig 4A**). A common origin is also predicted for the North American admixed strains (NoAm) as previously reported [22, 23, 25], where a PB-1 origin contributes to the genome of all isolates.

To infer the most likely parental subpopulations, genetic contribution to the admixed genomes and putative date of admixture, each admixed individual/subpopulation was analysed using GLOBETROTTER. In particular, GLOBETROTTER indicated that the admixed population of Karukinka (SoAm-3) also originated from an admixture event between PB1-Karukinka and PB3-Puyehue individuals, which contributed to 43% and 57% of the current genomes, respectively. Similarly, a pairwise nucleotide divergence (Dxy) analysis largely resembled the results obtained using cluster analysis (**Fig 4B**). Finally, to verify recent admixture events between lineages, a TreeMix analysis recapitulated independent events between subpopulations while fitting 8 migration events, consistent with the admixture analysis previously performed and depicting hybridization involving in all cases the PB-1 lineage (**Fig 4C**). Altogether, these results suggest recent outcrossing and admixture between *S*. *eubayanus* populations, where the PB-1 branch contributes to all the admixed genomes analysed in our study.

### Evidence of ancestral gene flow and admixture events between lineages

**Fig 5:**
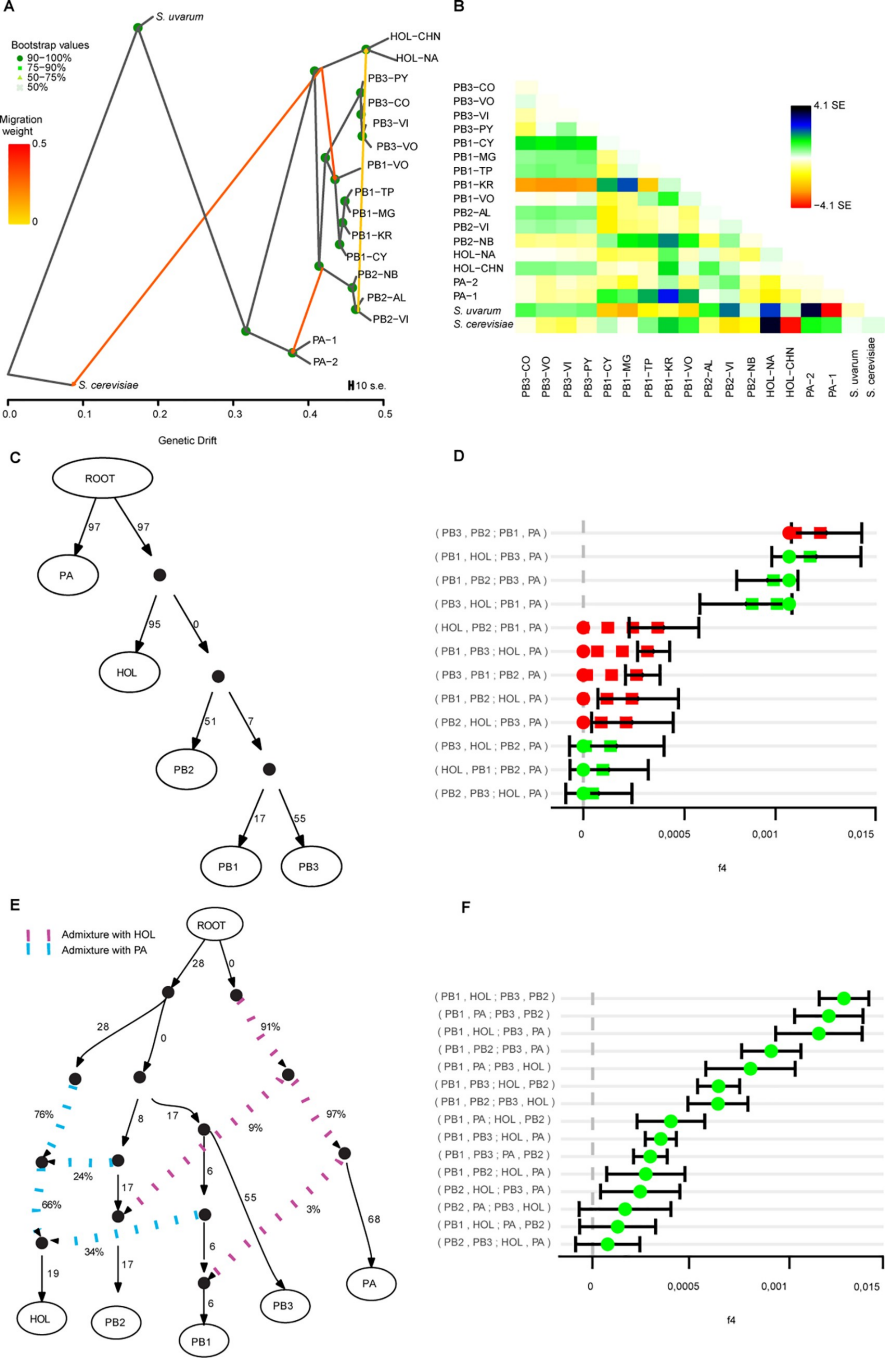
Admixture between ancestral *S*. *eubayanus* populations. (A) B) The residual matrix from the tree in A. (C) and (E) Admixture graphs of Model 1 (no admixture) and Model 3. Continuous lines indicate direct ancestry; numbers at these lines denote scaled drift (f2-statistic). Dotted lines indicate admixture; numbers at these edges show the percentage of ancestry contribution. (D) and (F) Goodness-of-fit of the graphs in C and E. Green and red dots indicate f4 values expected from the model. Red colour indicates deviance of the expected f4 value from the calculated f4 value (+/- stderr).

To identify population founders and historical hybridization events we applied explicit tests of admixture using TreeMix and f4-statistics together with admixture models using admixturegraph. In this analysis, none of the strains coming from recent admixture events were considered, and only strains belonging to the different lineages were used. The TreeMix topology without migrations (**S4A Fig and S4B Fig**) resembles our phylogenetic tree and explained 99.61% of the variance of the data. Adding, one, to six migration events increased the variance explained by the model from 99.88% to 99.97%, respectively. An Ad hoc analysis of the second order rate of change in the log-likelihood as migration events were added indicated two or four migration edges as the most likely models (**S5B Table**). The model that included four migration events, indicated gene flow between PB-1 –HOL and PB-2 and HOL ancestral lineages and the PA ancestor together with PB-2 (**Fig 5A and 5B**). Interestingly, gene flow between *S*. *cerevisiae* and the HOL lineage was found, suggesting additional signatures of ancestral hybridizations between both species, or a close sister species to *S*. *cerevisiae* (**S4C Fig and S4D Fig)**.

Next, we evaluated gene flow among *S*. *eubayanus* populations by calculating f4 statistics. As Treemix results suggested gene flow between *S*. *eubayanus* populations with different *Saccharomyces* sp, we did not use *S*. *uvarum* or *S*. *cerevisae* as outgroups. Instead, we calculated f4 statistics using only *S*. *eubayanus* populations and we tested whether f4 values agreed with *S*. *eubayanus* admixture models based on the phylogenetic tree topology, this is (PA,(HOL,(PB2,(PB1,PB3). We found a large discrepancy between calculated f4 values with the expected f4 values of a non-admixture model, suggesting the presence of admixture events between *S*. *eubayanus* populations (**Fig 5C and 5D**).

Next, we incorporated the three admixture events suggested by Treemix which improved the fit to f4 values (Model 2, **S4E Fig**, **S5C Table**), yet by adding one additional admixture event (PB-1—PA) we obtained Model 3 which had the lowest minimal error (0.000045, **Fig 5E and 5F**). In Model 3 ancestral populations of PB-1 and PB-2 had independent events of admixture with both, PA and HOL ancestral populations. Alternative models that could explain gene flow of the Patagonia B populations to either PA or HOL using fewer admixture events did not show a better fit (**S5D Table**). Altogether, our results demonstrate extensive gene flow and admixture between lineages. Furthermore, the current lineages do not correspond to the outcome of recent admixture events between any of the other lineages, in agreement with migration edges inferred by Treemix.

### Pangenome and gene content variation

To compare the genome content, we generated *de novo* assemblies and constructed the pangenome across all isolates (**S5E Table**). We identified 5,497 non redundant pangenomic ORFs in the species. Out of these, 5,233 ORFs are core systematically present in all of the isolates, while 264 are dispensable (**S5F Table**), being only found in subsets of strains. A PCA analysis of the presence/absence profile of core genes was used to visualize potential overlap between genome content similarities and SNPs distance (**S5A Fig**). In partial concordance with the phylogenetic tree, the branch harbouring the Chinese isolate, which also contained two North American isolates, represented the most divergent lineage, suggesting a recent migration event between Asia and America.

Overall, we were able to identify nine ORFs present in a group of nine closely related isolates belonging to the PB-2 lineage, representing a candidate lateral gene transfer (LGT) event from other specie(s). Interestingly, six of the strains were isolated from the same location, and these genes were assembled within a region of 7.3 kb on a single contig for each of the six isolates (*S*. *eubayanus* Region A, **S5B Fig**). The sequences of these contigs could not be found in the NCBI non-redundant database, with the exception of a 7 kb region, upstream to the group of ORFs, which matches the subtelomeric end of chromosome I. Without a match in another species, we were not able to confidently assign these genes to an LGT event. However, their subtelomeric localisation, together with their assembly on a single continuous region in a subset of isolates represent hallmarks of LGT identified in other *Saccharomyces* species [5, 27]. Subsequently, SMART (Simple Modular Architecture Research Tool) was used to identify known PFAM protein domains in these regions. We found several putative proteins, with domains such as: arginase domain, a membrane transport protein domain, a Fungal specific transcription factor domain, a Gal4-like dimerization domain and a transmembrane one (**S5C Fig** and **S5G Table**). In addition to these high-confidence hits, SMART identified the potential presence of a glucosidase domain and a homing endonuclease domain in four and two ORFs, respectively (**S5C Fig** and **S5G Table**).

Furthermore, we also identified 64 private ORFs in the Chinese/North American group of strains, where at least 27 (**S5H Table**) correspond to orthologs of other genes found in the lager *S*. *eubayanus* genome (showing an average sequence divergence of 12.4%). These features closely resemble those introgressions found from *S*. *paradoxus* into specific *S*. *cerevisiae* lineages [5]. However, no potential donor species was found and hence we cannot completely rule out different evolutionary origins, such incomplete lineage sorting and/or balancing selection.

### Phenotypic diversity and fermentation capacity among *S*. *eubayanus* isolates

**Fig 6:**
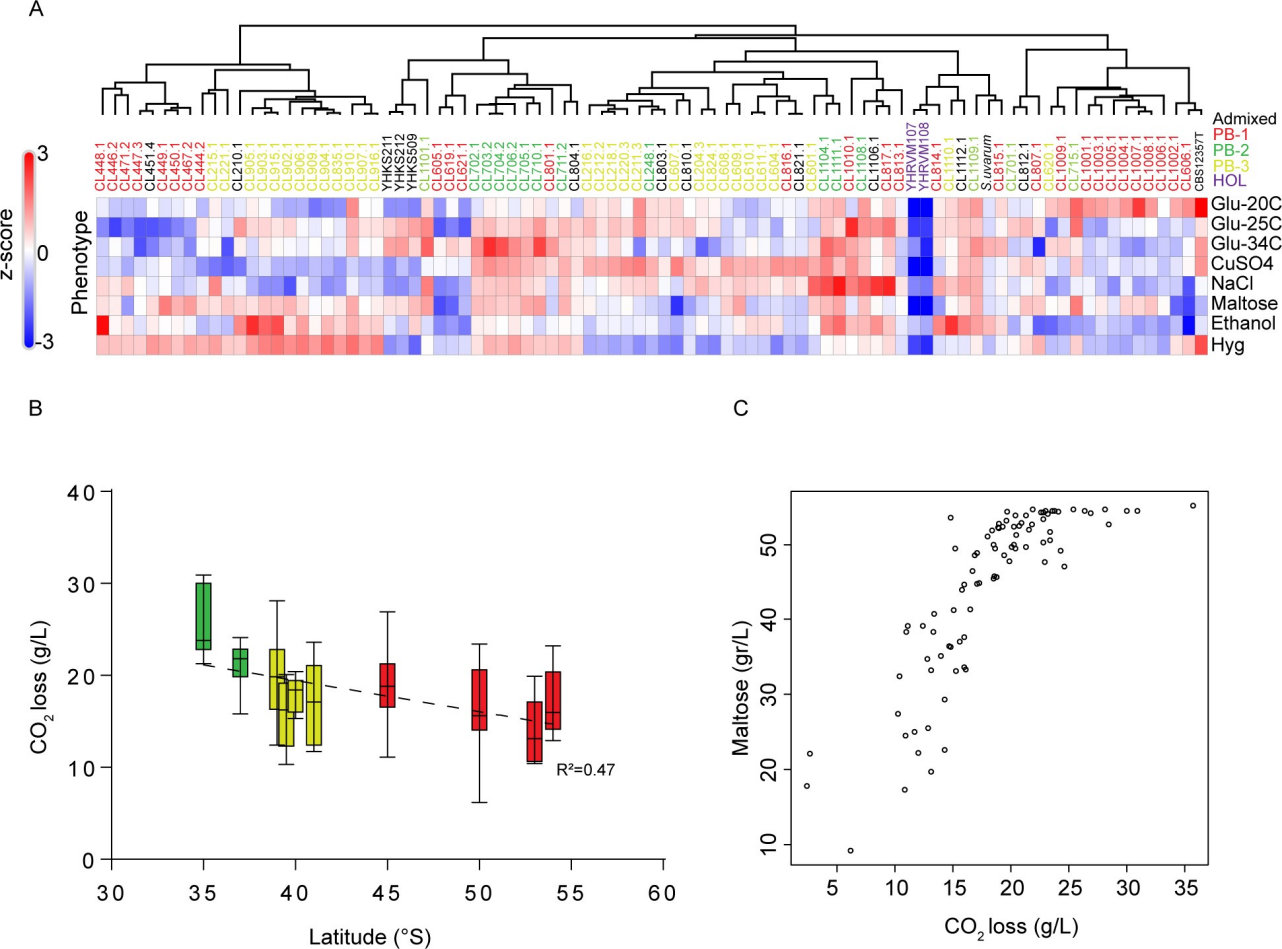
Phenotypic diversity in *S*. *eubayanus*. (A) Heat map depicting the phenotypic diversity in *S*. *eubayanus* obtained from eight different conditions assessed in microcultures. Strains are grouped by hierarchical clustering and names & colours indicate the lineage. The heat maps were elaborated based on z-scores within each phenotype. (B) CO_2_ loss levels represent the fermentative capacity of wild isolates obtained from microfermentations. Error bars denote the standard deviation (C) Maltose consumption was directly correlated with CO_2_ loss and latitude of the origin of the isolate.

*S*. *eubayanus* phenotypic diversity was assessed in a set of 89 isolates from North America (N = 5), and South America: Altos de Lircay (N = 9), Nahuelbuta (N = 10), Villarrica (N = 10), Choshuenco (N = 10), Puyehue (N = 6), Osorno Volcano (N = 6), Coyhaique (N = 9), Torres del Paine (N = 9), Magallanes (N = 5), Karukinka (N = 9), Argentina (N = 1) (**Fig 1A, S6A Table**). A clustered heat map of the phenotypic correlations between yeast isolates across all traits for μmax (corresponding to the maximum specific growth rate **S6B Table**, **Fig 6A**) showed that North American Holarctic strains clustered separately with low μmax and maxOD scores (maximum OD growth recorded) across traits, while the CBS12357^T^ strain clustered together with PB-1 strains from Torres del Paine. This clustering was further supported by the principal component analysis, both of which produced the main groups split by localities, rather than lineages, where only PB-2 isolates grouped together (**S6 Fig**). Specifically, only for certain phenotypes did we find differences among localities (**S6C Table**).

Subsequently, given the importance of *S*. *eubayanus* in lager brewing, we then evaluated the fermentation performance of the same set of strains previously phenotyped and used the *S*. *pastorianus* W34/70 control strain from lager as positive fermentation control. The conditions included 12°P wort at 12°C in 50 mL (micro-fermentations batches) and CO_2_ loss was recorded every day and metabolite consumption was estimated at the end of the fermentation process. In all cases, the lager control showed better fermentation performance compared to all *S*. *eubayanus* isolates (q*-*value < 0.05, **S7A Table**), yet several strains showed high CO_2_ release levels, demonstrating the beer fermentation potential of some strains. Overall, we observed that isolates obtained at lower latitudes (Central region) released significantly greater CO_2_ levels than individuals obtained at higher latitudes (extreme South, **S7B Table,** Pearson r = 0.566, *p*-value < 0.001, **Fig 6B**). Furthermore, we found that fermentation performance was directly correlated with maltose sugar consumption (Pearson r = 0.52, *p*-value < 6 x 10^−8^, **S7C Table, Fig 6C**), and ethanol production (Pearson r = 0.56, *p*-value < 2 x 10^−6^).

## Discussion

Our results, and those of other authors (see [19, 22]), strongly suggest that *S*. *eubayanus* preferentially associates with *Nothofagus* trees, particularly *N*. *pumilio*, the most cold-adapted member of this tree genus [28, 29]. Under this scenario, the biogeographical history of *S*. *eubayanus* should logically and strongly correlate with the history of *Nothofagus* dispersal across the globe. Interestingly, the isolation frequency of *S*. *eubayanus* was correlated with latitude. PB-1 is located at higher latitudes (and lower altitudes) and the isolation frequency of this lineage was lower than that of the other two major lineages, PB-2 and PB-3, which were easily recovered from the environment and were specifically associated with high altitude *N*. *pumilio* trees (**Fig 1**). The lower frequency of isolates found in samples from Tierra del Fuego could be due to the extreme environmental conditions found in this part of the continent, where average temperatures are below 5°C throughout most of the year [30]. However, the widespread distribution and higher abundance of *N*. *pumilio* in Tierra del Fuego compared to northern areas may facilitate the survival, range distribution, and habitat colonization of *S*. *eubayanus* [31], thus increasing its population size and genetic diversity. The isolation frequency of *S*. *eubayanus* in Patagonia was three times higher than that reported in China and North America, where a similar number of bark samples and sampling sites were surveyed [23–25]. Our results are also in agreement with those reported for *S*. *eubayanus* in Argentina, where high isolation rates in *N*. *pumilio* trees were also found [20]. The genus *Nothofagus* originated during the late Cretaceous-Early Tertiary interchange (ca. 135 MYA); between Southeast Asia and Australia, from a “fagalean” complex in Southeast Asia [28, 32]. At the time, Antarctica was in a more northern position connecting South America, Tasmania, Australia and New Zealand, and had a warm-humid climate (”mesothermal conditions”, sensu [28]). Subsequently, a step-wise wave of dispersion-colonization of *Nothofagus* in a westward direction reached South America. This scenario is consistent with a colonization of the *S*. *eubayanus* and *S*. *uvarum* ancestor across the South Hemisphere [23, 32].

The phylogenomic analysis presented here of wild *S*. *eubayanus* (**Fig 2**) reveals a higher genetic diversity for the PB South American lineages than that reported for *S*. *eubayanus* in the Northern Hemisphere [23, 25]. Indeed, our results resemble those obtained in the sister species for *S*. *uvarum*, where similar sequence divergence among *S*. *uvarum* populations was described [13], whereas in Chinese *S*. *cerevisiae*, a much higher genetic diversity is observed compared to other regions of the world [33]. Interestingly, based on the levels of genetic diversity and heterozygosity, our results support the idea that PB-1 *S*. *eubayanus* from Tierra del Fuego is the most represented population in Patagonia, and likely within the species (**Fig 2D**). This lineage shows high levels of hybridization/introgression into northern populations. The higher nucleotide diversity of PB-1 was supported by a trend of greater genetic diversity among individuals sampled from Coyhaique, Torres del Paine, Magallanes and Karukinka sites (**Fig 3B**), where the dispersal of *N*. *pumilio* is also greater than in more northern regions. However, since the three different lineages were geographically segregated, it is not possible to attribute increased genetic diversity exclusively to latitude or to the nature of PB-1, as all samples isolated at latitudes higher than 45°S were consistently assigned to PB-1. Nevertheless, our dense sampling procedure, together with other findings, suggest that other lineages are not present in the extreme south, therefore, we cannot determine whether the observed increment of genetic diversity with latitude is a general pattern [20, 23]. A subgroup of PB-1 isolates is found at lower latitudes, which means that PB-1, PB-2 and PB-3 are sympatric in northern regions. Furthermore, we found discrepancies between our genetic diversity estimates and previous reports [25], probably because only non-admixed isolates were used in our study, while previous datasets have utilized admixed genomes, inflating the estimated genetic diversity.

This scenario is also consistent with the idea that *S*. *eubayanus* cryotolerance evolved recently, as *N*. *pumilio* (its preferred environment) became secondarily adapted to cold during the orogenesis of the Andes [28, 29]. Also, it is now accepted that the distribution of *Nothofagus* (and subsequently *S*. *eubayanus*) was already established in Patagonia before the onset of the last glacial maximum (ca 20,000 years ago), when ice sheets covered most landmasses south of 43º. After this glacial period, there was massive floristic recolonization and ecological succession [29, 34]. Our results are consistent with this second scenario (colonization from peripheral glacial refugia from the South) since we found lower genetic diversity in populations located in central Chile (Altos de Lircay & Nahuelbuta) compared to populations found in southern Chile (Coyhaique, Torres del Paine, Magallanes and Karukinka). Interestingly, a different pattern is observed in the eastern side of the Andes (Argentina), where a lower number of peripheral glacial refugia occurred and most of the diversity would originate from Valleys refugia in northern sites [34]. Indeed, in Argentina there is greater *S*. *eubayanus* genetic diversity north of 43°, where different populations congregate in a single geographic location [23].

Our STRUCTURE analyses provide evidence of contact and admixture between PB-1 and the other two PB lineages (**Fig 2B**). Overall, all of the admixed strains from Chile, New Zealand and North America contained regions from the PB-1 lineage, that together with our population genetics analysis and admixture model between populations, suggest that PB-1 could be the most widespread lineage reported to date, including admixture with the Holarctic lineage. Indeed, higher *F*is values are found in the PB-1 cluster, compared to PB-2 and PB-3, suggesting that meiosis and inbreeding are more frequent in PB-1. Our admixture analysis demonstrates recurrent hybridization between ancestral lineages, supporting a scenario in which gene flow among Patagonian and Holarctic ancestral populations took place, and that is consistent with the co-occurrence of these populations in the past (**Fig 5**). Similar evidence of admixture in other yeast species, such as *S*. *cerevisiae*, *S*. *uvarum*, and *S*. *paradoxus* was previously reported [13–15]. Interestingly, we also detected evidence of ancestral gene flow between *S*. *cerevisiae* (or a close sister species) and the *S*. *eubayanus* HOL lineage. This demonstrates a pervasive contact between *Saccharomyces* populations and constant admixture, likely facilitated by their dispersal by insect’s in nutrient-rich periods of the year [16].

In summary, our results provide compelling evidence of the successful colonization and distribution of *S*. *eubayanus* in Patagonia, in the cold conditions of the southern hemisphere, representing most of the current extensive genetic diversity found in this species. The majority of the *S*. *eubayanus* strains collected around the world belong to the Patagonian cluster. This Out-of-Patagonia colonization has extended to the Northern Hemisphere, including a recently-found subset in China, and Oceania. Finally, our data concerning *S*. *eubayanus*, together with previous evidence in *S*. *uvarum* [13], leads us to propose that the ancestor of both species adapted to the environmental conditions in the Southern Hemisphere. However, the currently available data is insufficient to draw further conclusions regarding this claim, warranting future studies and especially experimental evidence for inferring local adaptation (e.g., fitness and common garden experiments, see [35]).

## Materials and methods

### Sample areas and yeast isolation

Bark samples from ‘lenga’ (*Nothofagus pumilio*), coigüe (*N*.*dombeyi*) and ‘ñirre’ (*N*. *Antarctica*) and *Araucaria araucana* were obtained aseptically from ten sampling sites in Chile (collection date, **Fig 1**): Sampling was performed as previously described [36]. Isolated colonies were stored in glycerol 20% v/v and stored at -80°C in the Molecular Genetics Laboratory yeast collection at Universidad de Santiago de Chile. Details on the localities and the yeast isolation procedures are available in S1 Text.

### *Saccharomyces eubayanus* identification and FACS analysis

We amplified and sequenced the internal transcribed spacer region (ITS) to identify colonies to the genus level [37]. *Saccharomyces* species identification was conducted using the polymorphic marker *GSY1* and *RIP1* through amplification and enzyme restriction (see details in [22]). In many cases, species identification was confirmed by Sanger-sequencing of the ITS region, which was attained using a BLASTN against the Genbank database under 100% identity as threshold. DNA content was analysed as previously described [5].

### Sequencing, Reads processing and Mapping

One isolate per tree was considered for DNA sequencing. DNA was obtained using a Qiagen Genomic-tip 20/G kit (Qiagen, Hilden, Germany). The library prep reaction used was a 100x miniaturized version of the Illumina Nextera method. Samples were sequenced on a NextSeq 500/550 High Output Kit v2.5 (300 Cycles) flow cell. Reads were processed with fastp 0.19.4 (-l 37–3) [38, 39]. Reads were aligned against the *Saccharomyces eubayanus* CBS12357^T^ reference genome [38] using BWA-mem [40].

### Variant calling

Mapping files were tagged for duplicates using Picard tools 2.18.14 (http://broadinstitute.github.io/picard/). Variant calling and filtering was done with GATK version 4.0.10.1 [41]. For all datasets, we only considered SNPs that had no missing data using vcftools option–max-missing 1. Furthermore, prior to making a dataset without missing SNPs, for gene flow analyses we excluded individuals that had more than 5% of missing sites, which included the lager strains, admixed strain yHKS212, and PB strains from Argentina. The effect of each variant was assessed and annotated with SnpEff version 4.3t [42], using an updated version of *S*. *eubayanus* gene annotations [38]

### Phylogeny and population structure analyses

To perform phylogeny on our SNP dataset, a VCF file containing 606,656 bialllelic SNPs was converted to phylip format and used as input for IQ-TREE [43] to generate a maximum likelihood phylogeny with the ultrafast bootstrap option and ascertain bias correction (-st DNA -o 1105.1_Nahuelbuta -m GTR+ASC -nt 8 -bb 1000)[44]. The number of parsimony informative sites was 156,051. Trees were visualized in the iTOL website (http://itol.embl.de). For STRUCTURE analysis, a thinned VCF file was generated with vcftools 0.1.15 (—thin 1000)[45], containing 9,885 similarly-spaced SNPs, while including only *S*. *eubayanus* strains. Structure was run five times (K ranging from 3 to 7), with 10,000 burn-in and 100,000 replications for each run. Optimal K values were obtained using structure-selector (http://lmme.qdio.ac.cn/StructureSelector/) [46] according to the Evanno method [47]. The resulting plots were obtained using CLUMPAK [48] and visualized using structure plot (http://omicsspeaks.com/strplot2/) [49]. We performed clustering analyses of the same samples by using SMARTPCA without outlier removal [50]. For fineSTRUCTURE analysis [51], a non-thinned VCF file was phased using BEAGLE 3.0.4 [52]. We used a constant recombination rate between consecutive SNPs based on *S*. *cerevisae* average recombination rate (0.4 cM/kbp, [53]). Chromosomal painting was performed with Chromopainter V2, and its output was further analysed with fineSTRUCTURE (-x 100000 -y 100000 -z 1000).

### Analyses of admixture

Historical and recent admixture between populations of *S*. *eubayanus* was tested with Treemix [54] and ADMIXTOOLS (**S2 Text**) [55]. For Treemix we analysed only *S*. *eubayanus* individuals that did not show any sign of recent admixture according to STRUCTURE results, plus the *S*. *uvarum* and a *S*. *cerevisiae* individual were kept as outgroups. In addition, we pruned out SNPs that were in linkage disequilibrium using PLINK (—indep-pairwise 50 10 0.2). We dissected the five *S*. *eubayanus* populations into subpopulations according to geographical locality and clusters obtained with fineSTRUCTURE as criteria. Treemix was first run ten times for each value of m (migration events) ranging from 1 to 6 (-noss–k 500) and two optimal m values (2 and 4) were estimated using the optM R package (https://cran.r-project.org/web/packages/OptM/index.html) (**S3 Table**). Treemix was subsequently run 100 times (2 and 4 migrations, -noss–k 500) after which a consensus tree and bootstrap values were obtained using the BITE R package [56]. We calculated f4 statistics between PA, PB-1, PB-2, PB-3, and HOL populations using the r package admixr [57]. Admixture graph fitting of the calculated f4 statistics was done using the R package admixturegraph [58]. Seven models were tested which were ranked according to their minimal error values (**S5D Table**).

### Population genetics

We estimated π and Tajima’s D using the R packages PopGenome 2.6.0 [59]. Values of *F*_st_ were calculated with StAMPP 1.5.1 Weir and Cockerham's unbiased estimator [60, 61] to obtain 95% confidence intervals by performing 5,000 bootstraps. LD decay was estimated by calculating R2 values using vcftools (--geno-r2 -ld-window -bp 100000), which were imported into R to calculate a regression according to [62], for which the half decay was estimated (Ldmax/2).

The variants of the *S*. *eubayanus* strains showing recent genetic admixture were split to bins of 100 SNPs and each bin was assigned to target populations using adegenet’s hyb.pred function [63]. GLOBETROTTER was run using the inputs generated for fineSTRUCTURE analyses (NULL IND = 0).

The R package hierfstat [64] was used to calculate *F*_is_, Hs, and Ho by using the basic.stats function. To perform a Mantel test, first the Nei’s genetic distances between subpopulations (considering localities) was calculated with the R package StAMPP [61].

### Pangenome

Isolates were assembled with Spades using k from 21 to 67. To detect the non-reference material we used the custom pipeline based on the method described in [5]. Potential lateral transferred ORFs were identified by blast search against an in-house database of 57 yeasts ORFeomes and to the currently available genomes from the yeast1000+ genome project (https://y1000plus.wei.wisc.edu/). SMART (Simple Modular Architecture Research Tool), used in GENOMIC mode, was used to identify known PFAM protein domains and homologies [65] using all the optional features: Outlier homologues, PFAM domains, signal peptides and internal repeats.

### Strains Phenotyping and Fermentations

The microcultivation phenotyping assay of the *S*. *eubayanus* strains was performed as previously described [66]. Briefly, isolates were pre-cultivated in 200 μL of YNB medium supplemented with glucose 2% for 48h at 25°C. For the experimental assay, strains were inoculated to an optical density (OD) of 0.03–0.1 (wavelenght 630 nm) in 200 μL of media and incubated without agitation at 25°C for 24 h (YNB control) and 48 h for other conditions in a Tecan Sunrise absorbance microplate reader. OD was measured every 20 minutes using a 630 nm filter. Each experiment was performed in quadruplicate. Maximum growth rate, lag time and OD max for each strain were calculated using GrowthRates software with default parameters [67]. Fermentations were conducted using a 12°P high-gravity wort at 12°C in 50 mL (micro-fermentations) using a Munton's Connoisseurs Pilsner Lager kit (Muntons plc, England). 50 mL of fresh wort were inoculated to a final concentration of 15 × 10^6^ viable cells/mL and fermentations were maintained for 14 days and weighed every day to calculate the CO_2_ output. At the end of the fermentation, metabolites were determined using HPLC.

## Supporting information

S1 Fig FACS analysis in *S*. *eubayanus isolates*. **(A)** Fluorescence values for each sample are shown in grey. (*red): haploid CL609.1; (*green): diploid CL1004.1; (*blue): tetraploid CL1005.1. (B) Number of cell vs propidium iodide intensity is shown. Haploid (n), diploid (2n) and tetraploid (4n) examples are shown for the same strains as above (*).
(PDF)

S2 Fig Co-ancestry matrix. A heatmap was obtained using fineSTRUCTURE chunkcounts. Each row and column represent an isolate and the colour scale indicates genetic sharing (yellow = low sharing, blue = high sharing). The tree shows the clusters inferred from the coancestry matrix. Populations and subpopulations can be inferred from the presence of darker colours in the diagonal
(PDF)

S3 Fig Tajima’s D and population differentiation values between lineages (*F*_ST_). (A) Tajima’s D values along the genome for PB-1 (top), PB-2 (middle) and PB-3 (bottom) lineages. Tajima’s D were estimated using the R packages PopGenome 2.6.0. (B). Individual example of extremely low Tajima’s D values in Chromosome V for PB-1 and PB-2. The close-up denotes the genes located within the low Tajima’s D region suggesting a common genetic ancestry.(B) *F*_ST_ between lineages. (C) Pairwise genetic distance between individuals versus geographic distance. Genetic distances were estimated using the Nei’s distance method. Geographic distances were estimated based on map coordinates in google maps (https://www.google.com/maps). A positive correlation between genetic distance and geographic distance was found.
(PDF)

S4 Fig Admixture models and gene flow between lineages. (A) Phylogenetic network obtained with Treemix allowing 0 (A) and 2 (C) migration events. Lines are coloured according to the migration weight which indicates the fraction of ancestry from the donor population. Arrows indicate the direction of the admixture event. The scale bar shows ten times the average standard error of the entries in the sample covariance matrix. (B) and (D) Residual fit of the trees in A and C respectively. (E) Admixture graphs of all models tested. Seven admixture graphs models were tested for their goodness of fit to f4 statistics. Continuous edges indicate direct ancestry; numbers at these edges denote scaled drift (f2-statistic). Dotted edges indicate admixture; numbers at these edges show the percentage of ancestry contribution. Graphs were constructed using the R package admixturegraph and were exported as qpgraph format.
(PDF)

S5 Fig Horizontal gene transfer event in PB-2. (A) Principal component analysis using only non-admixed 83 isolates from all populations shows partial concordance with the phylogenetic tree. The first component clearly separates the Chinese/North American branch from the South American lineages. Second component identifies the PB-3 as the most separated lineage, suggesting a lower level of outbreeding, while a partial overlap can be identified between the other lineages. The middle positioning of PB-1 using the third component mirrors the shape of the un-rooted phylogenetic tree based on the sequence divergence (Fig 2). (B) Nine ORFs, within the denominate Region A, have been identified on a single contig in 9 isolates. Around 6 kb of the flanking regions of these ORFs correspond to the chromosome I subtelomere, while the region where the ORFs are located do not show any similarities with known regions. (C) In the aminoacidic sequence of the nine ORFs, several domains can be identified of inferred by homologies.
(PDF)

S6 Fig Principal Component Analysis of growth rates obtained under eight different conditions across isolates. (PDF)

S1 Text Extended methods with details on the procedures utilised on each methods subsection. (DOCX)

S2 Text Detailed explanation of the admixture analyses performed. (DOCX)

S1 Table Number of samples obtained from each National Park. (XLSX)

S2 Table Bioinformatics Summary statistics together with NCBI accession numbers. (A) Bioinformatics Summary statistics and (B) Sequence identity matrix between strains.
(XLSX)

S3 Table Structure selector output. (XLSX)

S4 Table Population genetics summary statistics for each lineage & locality, Globetrotter analysis and *F*st values per lineages and localities. (A) Population genetics summary statistics for each lineage & locality, (B) Fst values.
(XLSX)

S5 Table Admixture test and f4 statistics. (A) Globetrotter analysis, (B) Optimal migration events for Treemix analysis, (C) f4 statistics, (D) Admixture graphs tested and their best error values, (E) de Novo assembly statistics, (F) Accessory genes in *S*. *eubayanus* and number of strains containing each gene, (G) Domains found in the candidate LGT in the PB-2 lineage, (H) Accessory ORFs found in the HOL lineage.
(XLSX)

S6 Table Phenotype data for *S*. *eubayanus* strains. (A). Raw phenotypic values. (B) Phenotype's correlations and (C). Phenotype comparison across localities.
(XLSX)

S7 Table Fermentation data for *S*. *eubayanus* strains. (A) CO_2_ lost in all strains. (B) Dunn's multiple comparisons test across localities and (C) Physical Chemical parameters after wort fermentation.
(XLSX)
