# Supplementary figures and images for: An Out-of-Patagonia migration explains the worldwide diversity and distribution of Saccharomyces eubayanus lineages
Source: PLoS Genet. 2020 May 1;16(5):e1008777. doi: 10.1371/journal.pgen.1008777 (PMC7219788; doi:10.1371/journal.pgen.1008777)

A

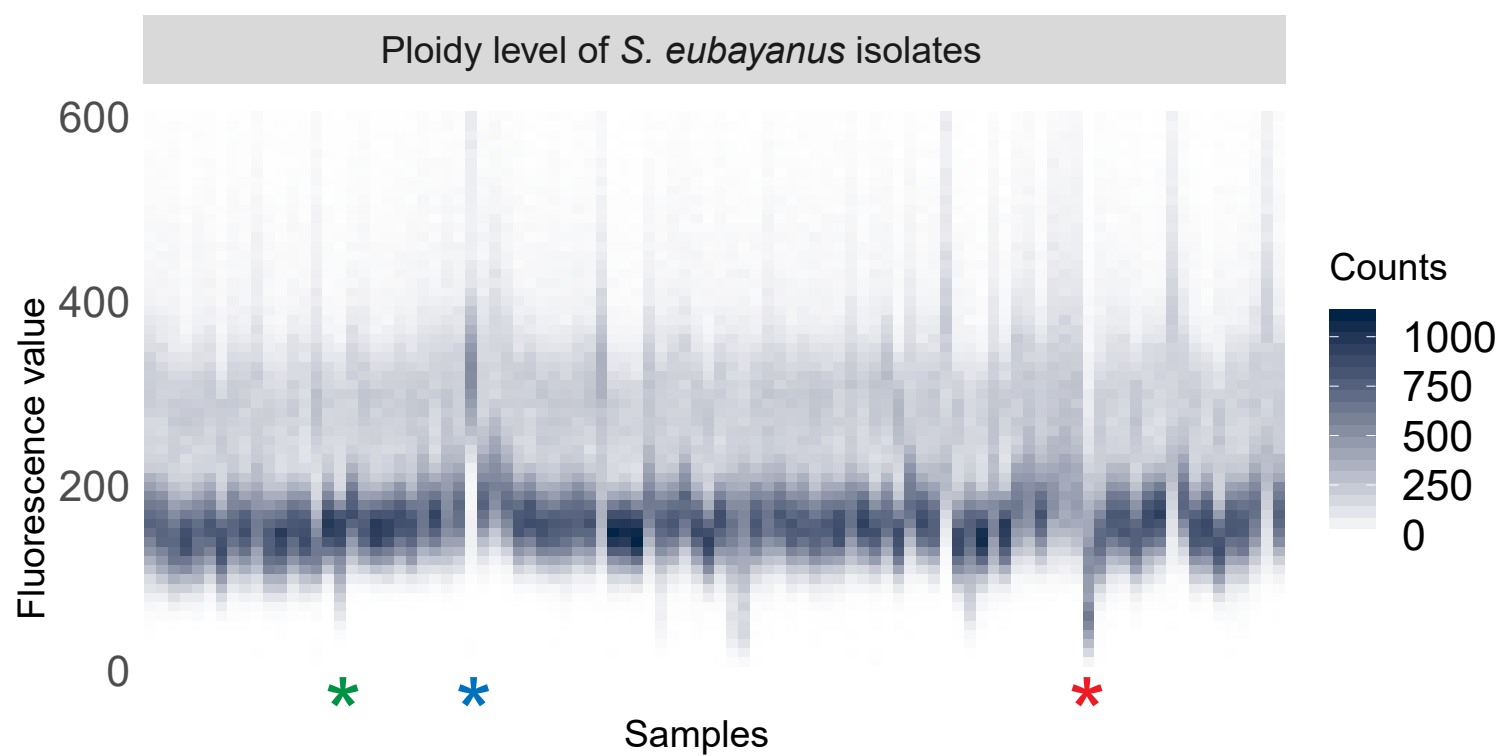

B

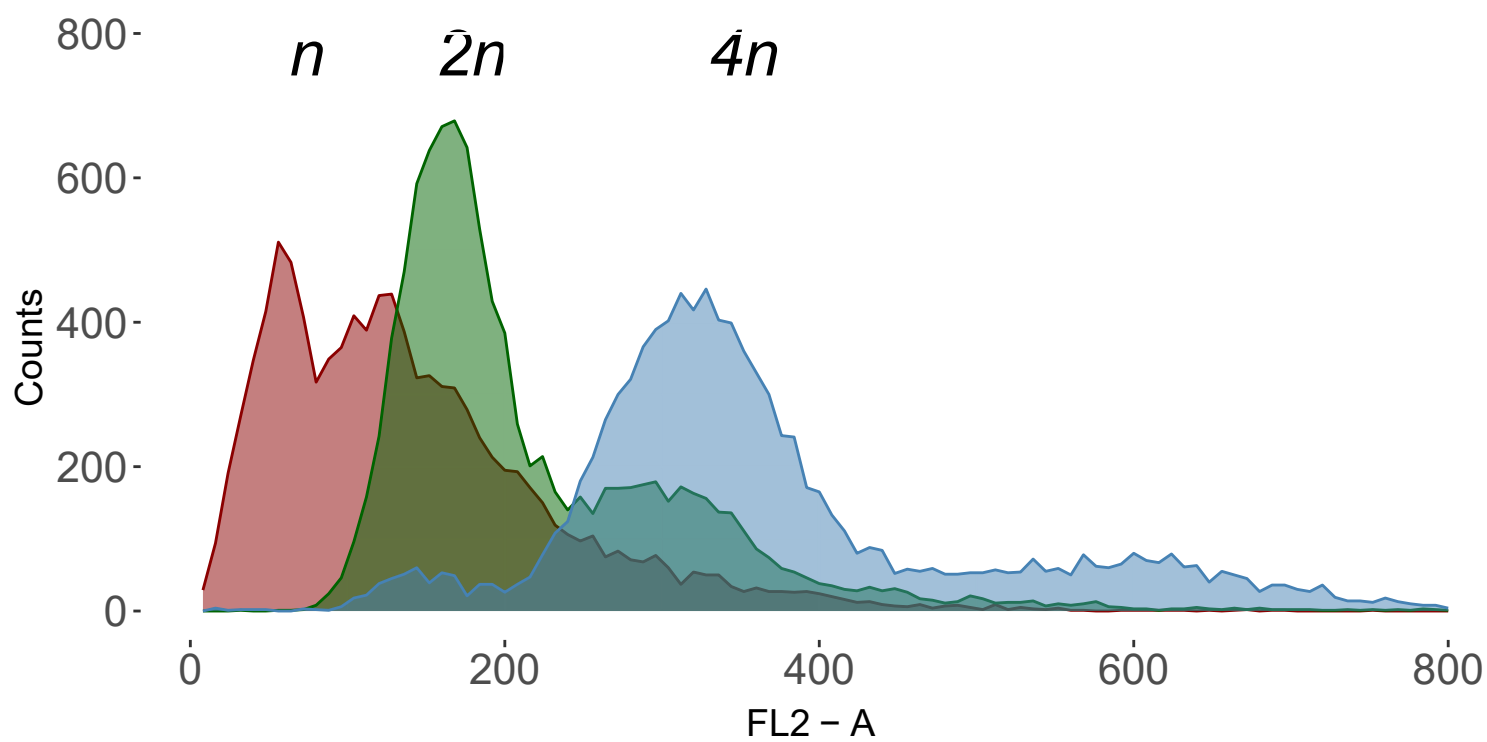

Figure S1

Supplement: S1 Fig — (A) Fluorescence values for each sample are shown in grey. (*red): haploid CL609.1; (*green): diploid CL1004.1; (*blue): tetraploid CL1005.1. (B) Number of cell vs propidium iodide intensity is shown. Haploid (n), diploid (2n) and tetraploid (4n) examples are shown for the same strains as above (*). (PDF) [file pgen.1008777.s001.pdf]

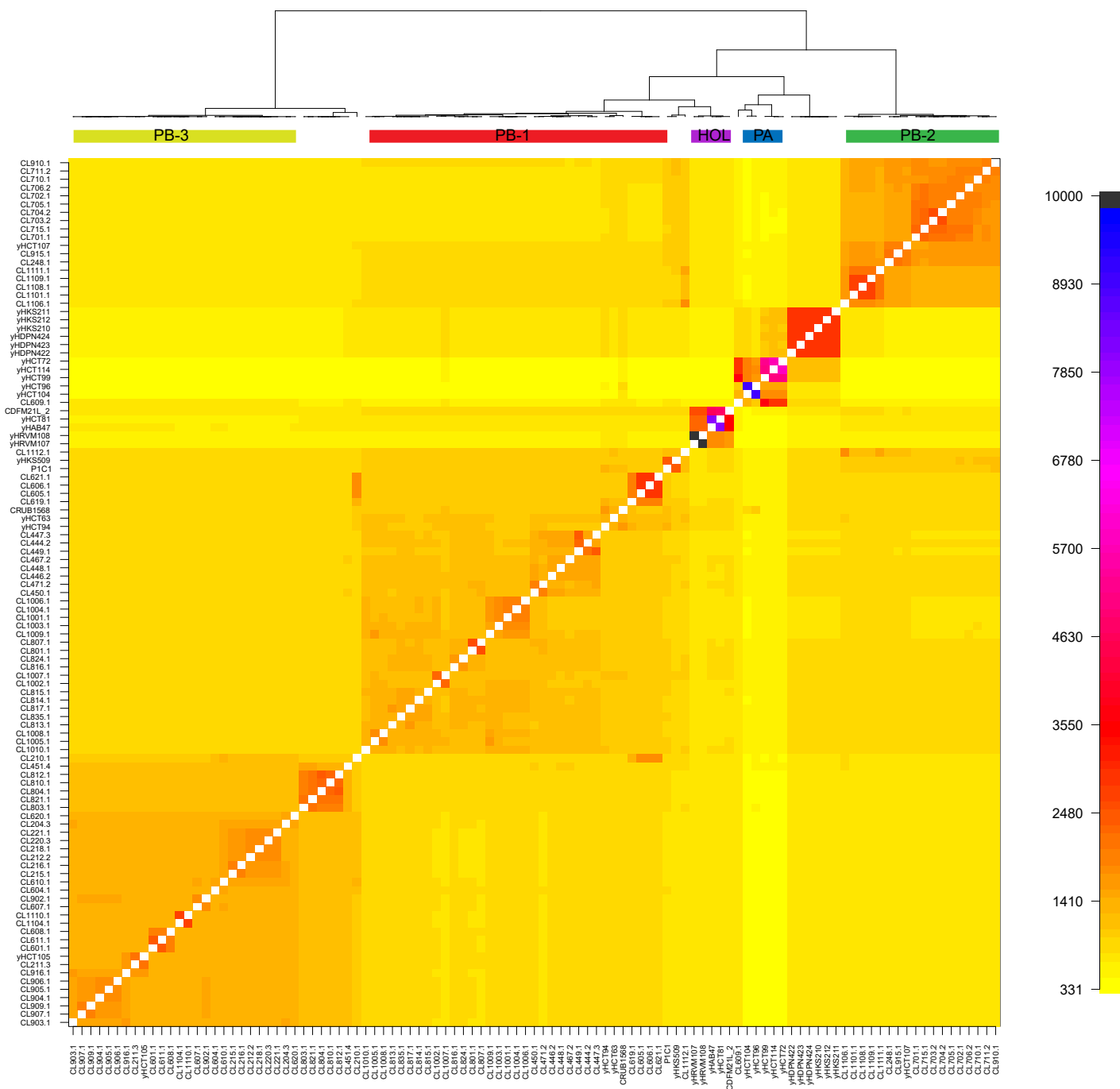

## Figure S2

Supplement: S2 Fig — A heatmap was obtained using fineSTRUCTURE chunkcounts. Each row and column represent an isolate and the colour scale indicates genetic sharing (yellow = low sharing, blue = high sharing). The tree shows the clusters inferred from the coancestry matrix. Populations and subpopulations can be inferred from the presence of darker colours in the diagonal (PDF) [file pgen.1008777.s002.pdf]

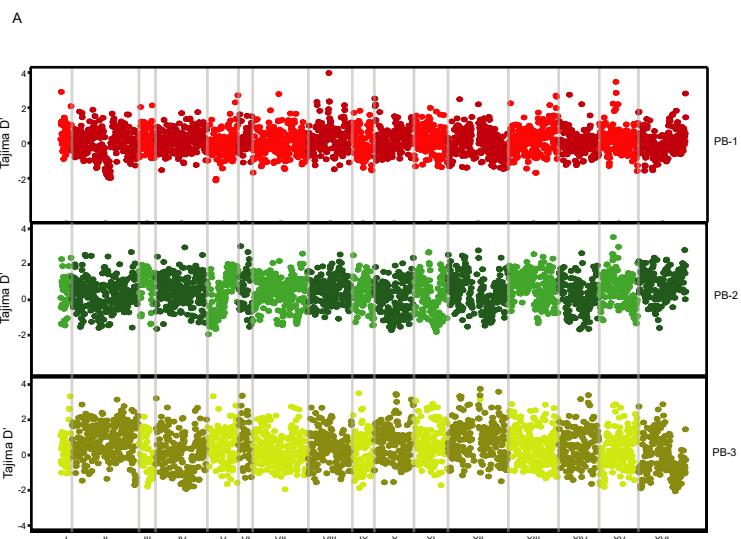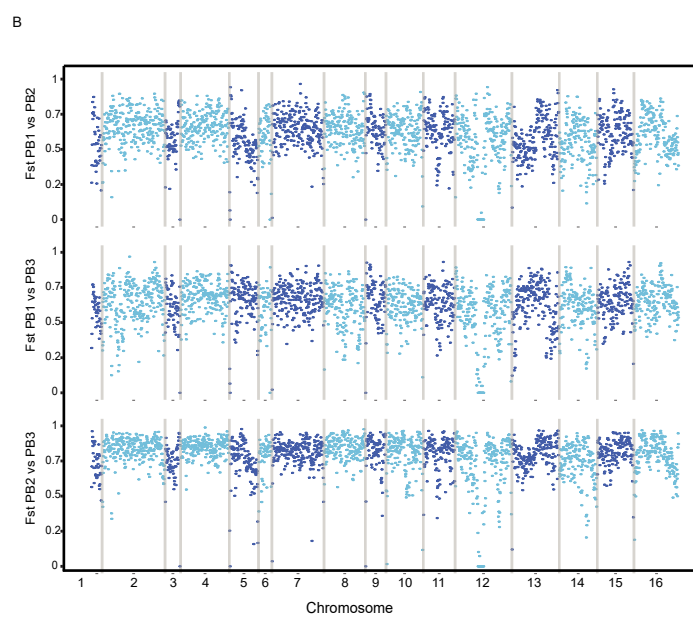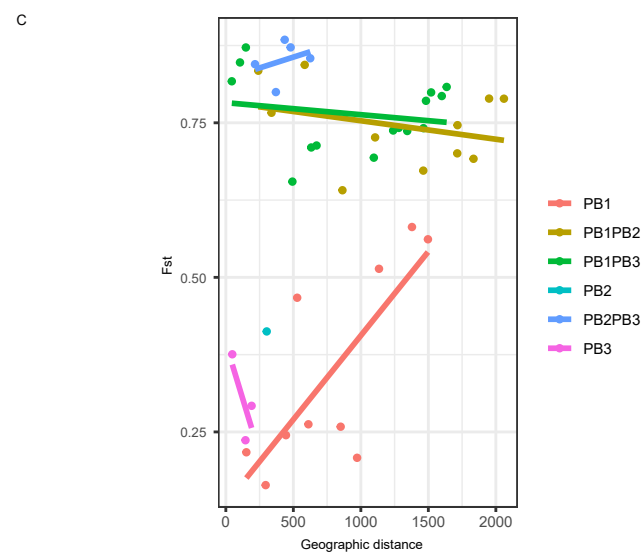

Figure S3

Supplement: S3 Fig — (A) Tajima’s D values along the genome for PB-1 (top), PB-2 (middle) and PB-3 (bottom) lineages. Tajima’s D were estimated using the R packages PopGenome 2.6.0. (B). Individual example of extremely low Tajima’s D values in Chromosome V for PB-1 and PB-2. The close-up denotes the genes located within the low Tajima’s D region suggesting a common genetic ancestry.(B) FST between lineages. (C) Pairwise genetic distance between individuals versus geographic distance. Genetic distances were estimated using the Nei’s distance method. Geographic distances were estimated based on map coordinates in google maps (https://www.google.com/maps). A positive correlation between genetic distance and geographic distance was found. (PDF) [file pgen.1008777.s003.pdf]

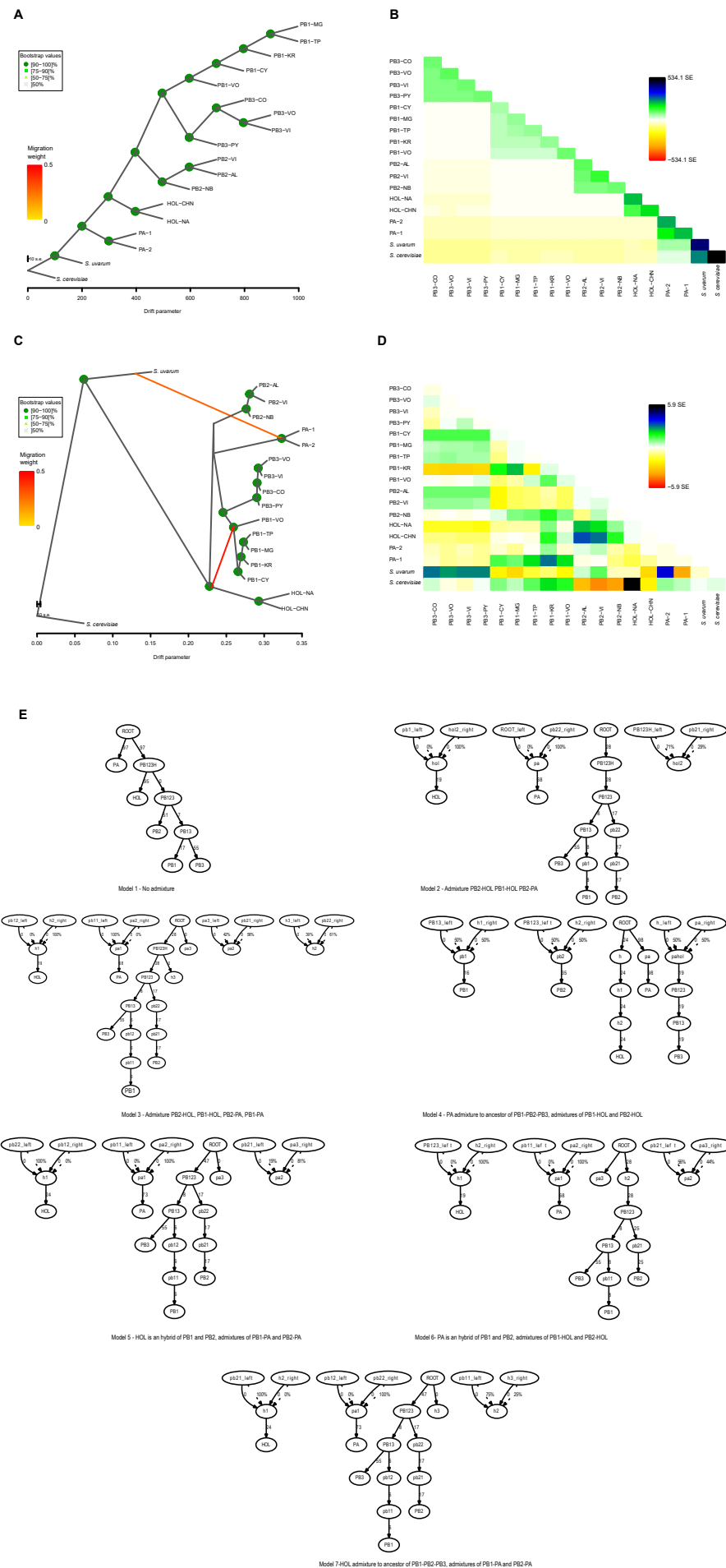

Figure S4.

Supplement: S4 Fig — (A) Phylogenetic network obtained with Treemix allowing 0 (A) and 2 (C) migration events. Lines are coloured according to the migration weight which indicates the fraction of ancestry from the donor population. Arrows indicate the direction of the admixture event. The scale bar shows ten times the average standard error of the entries in the sample covariance matrix. (B) and (D) Residual fit of the trees in A and C respectively. (E) Admixture graphs of all models tested. Seven admixture graphs models were tested for their goodness of fit to f4 statistics. Continuous edges indicate direct ancestry; numbers at these edges denote scaled drift (f2-statistic). Dotted edges indicate admixture; numbers at these edges show the percentage of ancestry contribution. Graphs were constructed using the R package admixturegraph and were exported as qpgraph format. (PDF) [file pgen.1008777.s004.pdf]

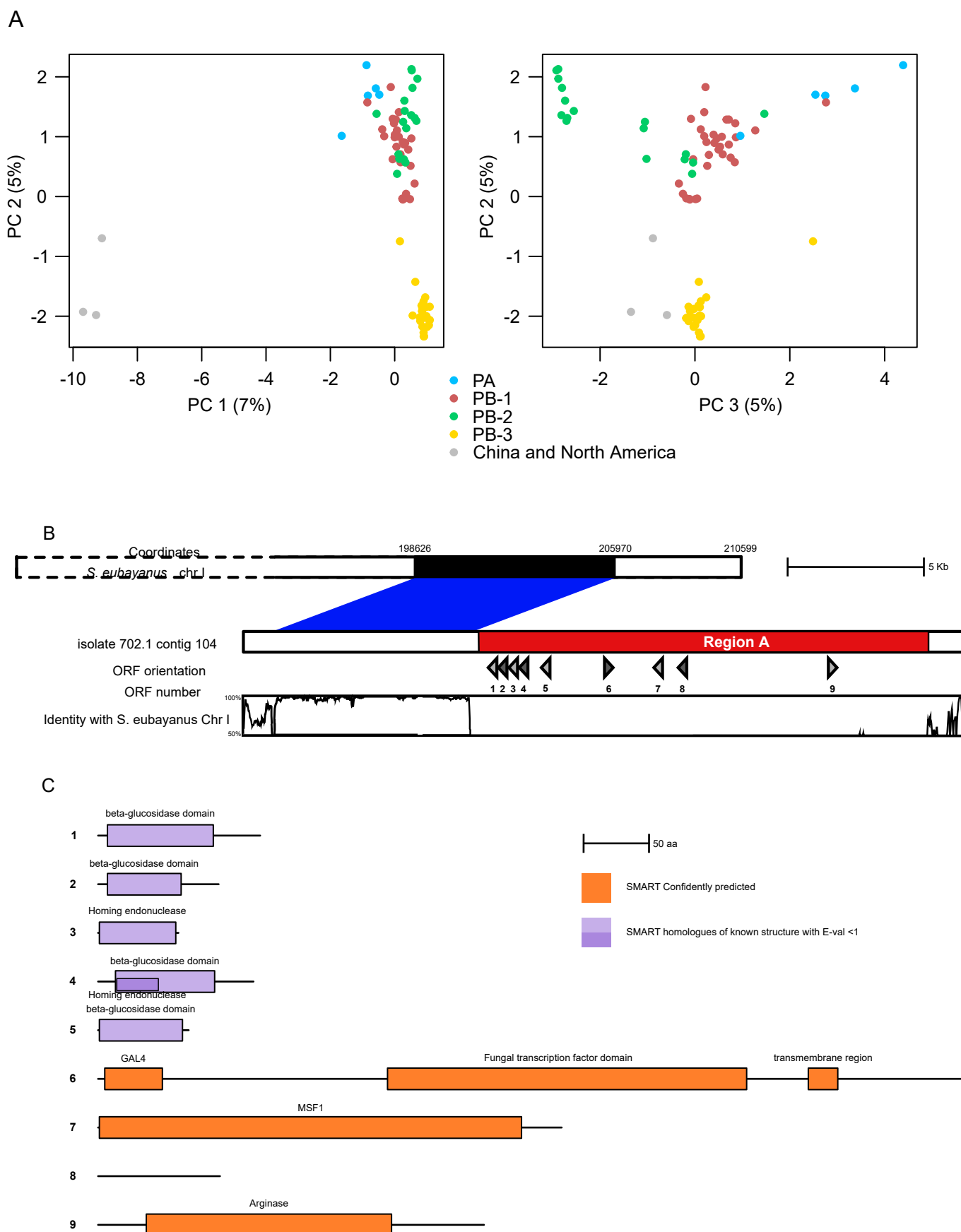

Figure S5

Supplement: S5 Fig — (A) Principal component analysis using only non-admixed 83 isolates from all populations shows partial concordance with the phylogenetic tree. The first component clearly separates the Chinese/North American branch from the South American lineages. Second component identifies the PB-3 as the most separated lineage, suggesting a lower level of outbreeding, while a partial overlap can be identified between the other lineages. The middle positioning of PB-1 using the third component mirrors the shape of the un-rooted phylogenetic tree based on the sequence divergence (Fig 2). (B) Nine ORFs, within the denominate Region A, have been identified on a single contig in 9 isolates. Around 6 kb of the flanking regions of these ORFs correspond to the chromosome I subtelomere, while the region where the ORFs are located do not show any similarities with known regions. (C) In the aminoacidic sequence of the nine ORFs, several domains can be identified of inferred by homologies. (PDF) [file pgen.1008777.s005.pdf]

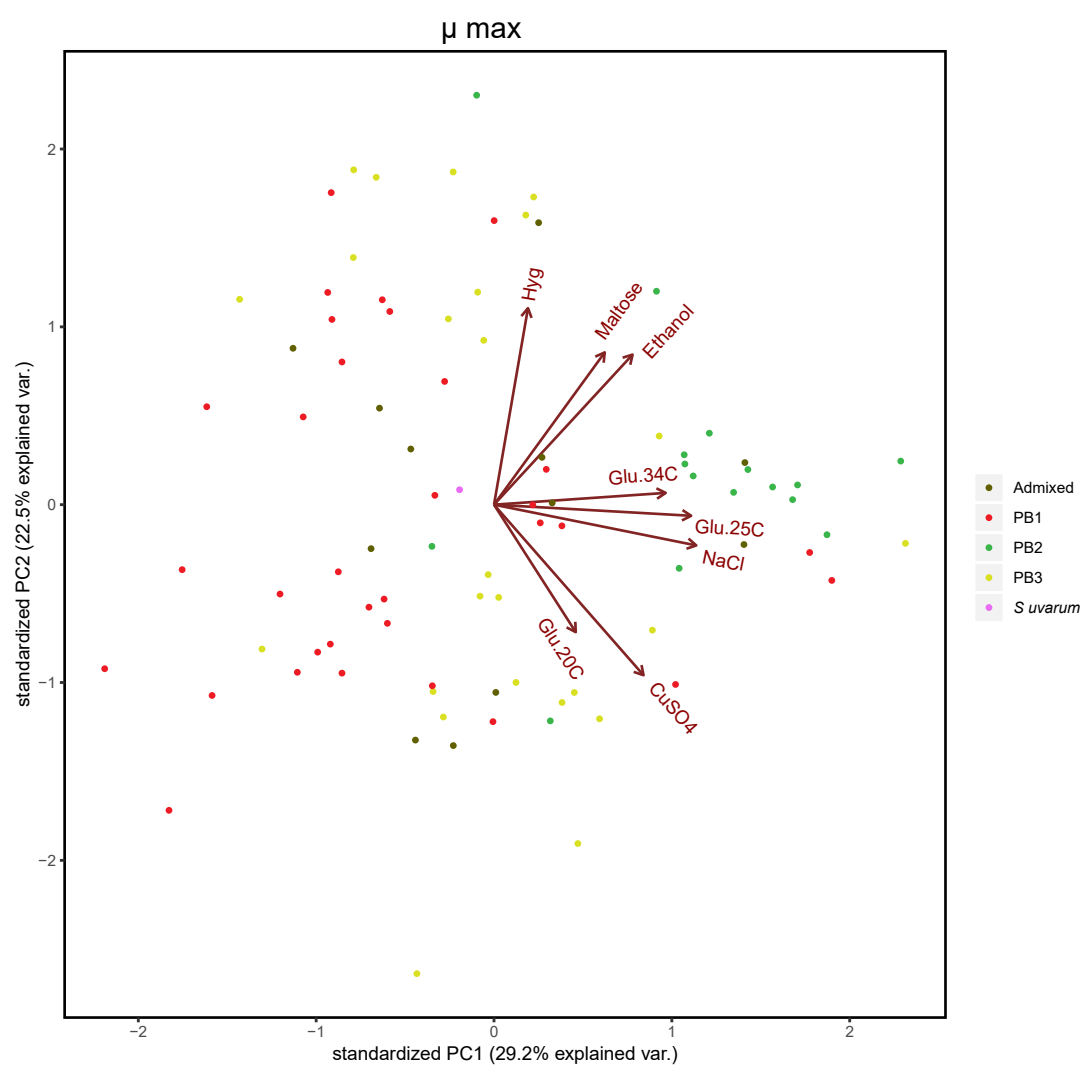

Figure S6

Supplement: S6 Fig — (PDF) [file pgen.1008777.s006.pdf]
