# Supplementary material for: An Out-of-Patagonia migration explains the worldwide diversity and distribution of Saccharomyces eubayanus lineages
Source: PLoS Genet. 2020 May 1;16(5):e1008777. doi: 10.1371/journal.pgen.1008777 (PMC7219788; doi:10.1371/journal.pgen.1008777)
Supplement: S1 Text — (DOCX) [file pgen.1008777.s007.docx]

**Supplementary Methods**

*Sample areas and yeast isolation*

Bark samples from ‘lenga’ (*Nothofagus pumilio*), coigüe (*N.dombeyi*) and ‘ñirre’ (*N. Antarctica*) and *Araucaria araucana* were obtained aseptically from ten sampling sites in Chile (collection date, GPS coordinates, **Figure 1**): National Park Altos de Lircay (January 2018, 35°36’34’’S, 70°57’58’’W), Nahuelbuta National Park (February 2018, 37°47’33’’S, 72°59’53’’W), Villarrica National Park (January 2017, 39°28’52’’S, 71°45’50’’W), Choshuenco National Park (January 2018, 39°50’2’’S, 72°4’57’’W), Antillanca National Park (November 2017, 40°46’23’’S, 72°12’15’’W), Vicente Pérez Rosales National Park (November 2017, 41°6’15’’S, 72°29’45’’W), Coyhaique National Reserve (February 2017, 45°31’23’’S, 71°59’19’’W), Torres del Paine National Park (February 2018, 50°56’32’’S, 73°24’24’’W), Magallanes National Reserve (January 2018, 53°8’45’’S, 71°0’12’’W) and Karukinka Natural Park (January 2018, 54°6’4’’S, 69°21’24’’W). All sampling sites were located at least five km from human settlements.

For each site, at least 25 bark samples of about 1g and 20 x 1 mm were obtained and immediately incubated in a 15 mL tube containing 10 mL of enrichment media. The media contained 2% yeast nitrogen base, 1% raffinose, 2% peptone and 8% ethanol [1]. Overall, 553 samples were collected (**Table S1**). Samples were incubated for two weeks at 20°C without agitation and were subsequently vortexed and plated (5 μL) onto YPD agar (1% yeast extract, 2% peptone, 2% glucose and 2% agar). Isolated colonies were stored in glycerol 20% v/v and stored at -80°C in the Molecular Genetics Laboratory yeast collection at Universidad de Santiago de Chile.

*Saccharomyces eubayanus* identification *and FACS analysis*

We amplified and sequenced the internal transcribed spacer region (ITS) to identify colonies to the genus level. For this, ITS1 and ITS4 primers [2] were used and we classified as *Saccharomyces* fragment sizes ranging between 830 and 880 bp [3]. Species identification was conducted using the polymorphic marker *GSY1* and *RIP1* through amplification and enzyme restriction (see details in [4]). Then, restriction fragment length polymorphism was performed using the restriction enzymes *HaeIII* and *EcoRI* as previously described [4]. Colonies were classified based on restriction patterns as either *S. eubayanus*, *S. uvarum* or *S. cerevisiae* [4]. In many cases, species identification was confirmed by Sanger-sequencing of the ITS region, which was attained using a BLASTN against the Genbank database under 100% identity as threshold.

DNA content was analysed using a propidium iodide (PI) staining assay. Cells were first pulled out from glycerol stocks on YPD solid media and incubated overnight at 30 °C. The following day a small portion of each patch was taken with a pipette tip and transferred in liquid YPD in a 96-well plate and incubated overnight at 30 °C. Then, 3 μl were taken and resuspended in 100 μl of cold 70% ethanol. Cells were fixed overnight at 4 °C, washed twice with PBS, resuspended in 100 μl of staining solution (15 μM PI, 100 μg/ml RNase A, 0.1% v/v Triton-X, in PBS) and finally incubated for 3 h at 37 °C in the dark. Ten thousand cells for each sample were analysed on a FACS-Calibur flow cytometer using the HTS module for processing 96-well plates. Cells were excited at 488 nM and fluorescence was collected with a FL2-A filter. The data collected were analysed in R with flowCore [5] and flowViz [6] and plotted with ggplot. The highest density value of FL2-A was associated with the ploidy level of G1 cells, thus cells that are not dividing, and used for inferring the ploidy state of the sample. FL2-A values between 60 and 110 for G1 cells were associated with haploid state, FL2-A values between 120 and 220 were associated with diploid state and FL2-A values between 290 and 400 were associated with a tetraploid state.

*Sequencing, Reads processing and Mapping*

DNA was obtained using a Qiagen Genomic-tip 20/G kit (Qiagen, Hilden, Germany). The library prep reaction used was a 100x miniaturized version of the Illumina Nextera method. In this prep, 1.6 ng of total DNA mass is tagmented in a 5X diluted Tagmentation reaction. The 0.5 μL reaction was quenched by 0.5% SDS(0.125% final concentration) at room temperature for 5 minutes. After quenching, 125 nL of a P5 sequencing barcode and 125 nL of a P7 sequencing barcode were added to the 0.625 nL reaction. In order to amplify the library of inserts, 24.125 μL of 1X KAPA Library Amplification Master Mix were added to the reaction. The library went through 15 cycles of PCR to add the barcodes to then amplify the library to a concentration >4 nM. The libraries were then normalized and pooled according to the Illumina standard operating procedure and sequenced on a NextSeq 500/550 High Output Kit v2.5 (300 Cycles) flow cell.

Read quality and summary statistics were examined using FastQC 0.11.8 [7]. Reads were processed with fastp 0.19.4 (low quality 3’ end trimming, 37 bp minimum read size) [8, 9]. We also obtained publicly available sequencing reads of *S. eubayanus* [8, 10-12] and *S. pastorianus* (Baker et al, 2015} from the SRA database, which were processed similarly, i.e. visual inspection with FastQC and processing adaptors, low quality 3’ ends, and read size, with fastp. Processed reads were aligned against the *Saccharomyces eubayanus* CBS12357^T^ reference genome [8] using BWA-mem (options: -M -R)[13]. Mapping quality and overall statistics were collected and examined with Qualimap [14]. Summary statistics are shown in **Table S2**. Sorting and indexing of output bam files were performed using SAMTOOLS 1.9 [15]. A *S. uvarum* isolate (CL1105) isolated from Nahuelbuta was also mapped to the *S. eubayanus* and *S. uvarum* CBS7001 genome [16, 17] for phylogenetic analysis. In addition, for Treemix analysis, *S. cerevisiae* reads (PRJNA340312) were mapped to *S. eubayanus* genome.

*Variant calling*

Mapping files were tagged for duplicates using MarkDuplicates of Picard tools 2.18.14 (http://broadinstitute.github.io/picard/). Variant calling and filtering was done with GATK version 4.0.10.1 [18]. More specifically, variants were called per sample and chromosome using HaplotypeCaller (default settings), after which variant databases were build using GenomicsDBImport. Genotypes for each chromosome were called using GenotypeGVCFs (-G StandardAnnotation). Variant files were merged into one genome-wide file using MergeVcfs. This file was divided by SNP calls and INDEL calls using SelectVariants. We applied GATK recommended filters to both variant files, i.e. for SNPs “QD < 2.0 || FS > 60.0 || MQ < 40.0 || MQRankSum < -12.5 || ReadPosRankSum < -8.0”, and for INDELS “QD < 2.0 || FS > 200.0 || ReadPosRankSum < -20.0". Furthermore, we applied a stricter criteria to filter heterozygous calls using bcftools view (-e 'GT="0/1" & QUAL<7000 & AC=1') version 1.9 [15]}. This VCF file was further filtered, depending of what was required for the given analysis, using vcftools [19]. For all datasets, we only considered SNPs that had no missing data using vcftools option –max-missing 1. Furthermore the effect of each variant was assessed and annotated with SnpEff version 4.3t [20], using an updated version of *S. eubayanus* gene annotations [8]

*Phylogeny and population structure analyses*

To perform phylogeny on our SNP dataset, a VCF file containing 606,656 bialllelic SNPs was converted to phylip format and used as input for IQ-TREE [21] to generate a maximum likelihood phylogeny with the ultrafast bootstrap option and ascertain bias correction (-st DNA -o 1105.1_Nahuelbuta -m GTR+ASC -nt 8 -bb 1000) [22]. The number of parsimony informative sites were 156,051. Trees were visualized in the iTOL website (http://itol.embl.de). For STRUCTURE analysis, a thinned version of the VCF file was generated with vcftools 0.1.15 (--thin 1000)[23], containing 9,885 similarly-spaced SNPs, while including only *S. eubayanus* strains. Structure was run on this dataset five times for K values ranging from 3 to 7, with 10,000 burn-in and 100,000 replications for each run and using admixture model, infer alpha, lambda =1, fpriormean =1, unifprioralpha 1, alpha max 10. The structure-selector website was used to obtain the optimal K values (http://lmme.qdio.ac.cn/StructureSelector/) [24] according to the Evanno method [25] and to obtain the final results for each K, which were plotted using CLUMPAK [26]. The resulting diagrams were visualised using structure plot (http://omicsspeaks.com/strplot2/)[27]. In addition, we performed clustering analyses of the same samples by using SMARTPCA without outlier removal [28]. For fineSTRUCTURE analysis [29], a VCF file that included all SNPs called among *S. eubayanus* strains was phased using BEAGLE 3.0.4 [30]. As we lacked a *S. eubayanus* recombination map, we used a constant recombination rate between consecutive SNPs based on *S. cerevisae* average recombination rate (0.4 cM/kbp, [31]). All versus all chromosomal painting was performed with Chromopainter V2, and its output was further analysed with fineSTRUCTURE (-x 100000 -y 100000 -z 1000). Plotting of the ancestry matrix was done using fineSTRUCTURE R scripts.

*Analyses of ancient and recent admixture*

Ancient admixture between populations of *S. eubayanus* was tested with Treemix [32] and ADMIXTOOLS [33]. For Treemix we analysed only *S. eubayanus* individuals that did not show any sign of recent admixture according to STRUCTURE results, plus the *S. uvarum* and a *S. cerevisiae* individual were kept as outgroups. In addition, we pruned out SNPs that were in linkage disequilibrium using PLINK (--indep-pairwise 50 10 0.2). We dissected the five *S. eubayanus* populations into subpopulations according to geographical locality and clusters obtained with fineSTRUCTURE as criteria. Treemix was first run ten times for each value of m (migration events) ranging from 1 to 6 (-noss –k 500) and two optimal m values (2 and 4) were estimated using the optM R package (<https://cran.r-project.org/web/packages/OptM/index.html>) (Table S3a). Treemix was subsequently run 100 times (2 and 4 migrations, -noss –k 500) after which a consensus tree and bootstrap values were obtained using the BITE R package [34]. We calculated f4 statistics between PA, PB-1, PB-2, PB-3, and HOL populations using the r package admixr [35]. Admixture graph fitting of the calculated f4 statistics was done using the R package admixturegraph [36]. Seven models were tested which were ranked according to their minimal error values.

The variants of the mosaic *S. eubayanus* strains were split to bins of 100 SNPs (on average ~5kb windows) and each bin was assigned to either of the populations (i.e. PB-1, PB-2, PB-3, or PA) using adegenet’s hyb.pred function [37] . This algorithm uses DAPC to estimate membership probability of a hybrid dataset to a known cluster (populations). Another sliding window analysis was used to calculate nucleotide divergence across the genome of the mosaic strains against their most likely parental subpopulations by using PopGenome’s “diversity stats between” analysis on bins of 100 SNPs (version 2.6.0) [38]. We used GLOBETROTTER [39] to estimate the most likely parent subpopulations and the generations since admixture of the mosaic strains. GLOBETROTTER was run using the output of Chromopainter V2 which this time was performed using the target and candidate donor subpopulations (NULL IND = 0). Mosaic strains of Karukinka and North America were grouped as populations which allowed us to bootstrap the value of their admixture event date (20 bootstraps). TREEMIX was also ran using a LD-filtered version of this dataset while allowing for 8 migration events. For all gene flow analyses, we excluded individuals that had more than 5% of missing data, which included the lager strains, and PB strains from Argentina.

*Population Genetics*

We estimated π and Tajima’s D using the R packages PopGenome. Values of *F*_st_ were calculated with StAMPP 1.5.1 Weir and Cockerham's unbiased estimator [40, 41] to obtain 95% confidence intervals by performing 5,000 bootstraps. LD decay was estimated by calculating R2 values using vcftools (----geno-r2 --ld-window-bp 100000), which were imported into R to calculate a regression according to [42], for which the half decay was estimated (Ldmax/2).

The R package hierfstat [43] was used to calculate *F*_is_, Hs, and Ho by using the basic.stats function. Bootstrapping per loci on each population’s *F*_is_ was done using hierfstat’s boot.ppfis, obtaining the 50th and 97.5th quantiles after 50000 boostraps. To perform a Mantel test, first the Nei’s genetic distances between subpopulations (considering localities) was calculated with the R package StAMPP [41]. Euclidean distance between localities was calculated using latitude and distances coordinates with R ‘dist’ function. Randel test was performed using the ade4 R package [44].

*Pangenome*

Isolates were assembled with Spades using k from 21 to 67. To detect the non-reference material, we used the custom pipeline based on the method described in [45]. LRSDAY software [46] was used to annotate the non-reference material. The newly annotated ORFs were added to the reference ORFs and a custom pipeline, also based on methods from [45] was used to collapse ORFs with identity percentage over 95, selecting an unique reference for each groups of allelic variants to obtain a list of non-redundant pangenomic ORF sequences. Confirmation of presence of these ORFs was obtained by mapping the reads of each strain to the set of pangenomic ORFs using BWA mem with the option – U 0. Filtering was performed with samtools with options –bSq 20 –F260. To identify potential lateral transferred ORFs we blast searched against an in-house database of 57 yeasts ORFeomes and to the currently available genomes from the yeast1000+ genome project (https://y1000plus.wei.wisc.edu/). To be robust, a LGT hit should cover over 75% of the query with an id >90%. SMART (Simple Modular Architecture Research Tool), used in GENOMIC mode, was used to identify known PFAM protein domains and homologies [47] using all the optional features: Outlier homologues, PFAM domains, signal peptides and internal repeats.

*Strains Phenotyping* *and Fermentations*

The microcultivation phenotyping assay of the *S. eubayanus* strains was performed as previously described [48]. Briefly, isolates were pre-cultivated in 200 μL of YNB medium supplemented with glucose 2% for 48h at 25°C. For the experimental assay, strains were inoculated to an optical density (OD) of 0.03–0.1 (wavelenght 630 nm) in 200 uL of media and incubated without agitation at 25°C for 24 h (YNB control) and 48 h for other conditions in a Tecan Sunrise absorbance microplate reader. OD was measured every 20 minutes using a 630 nm filter. Each experiment was performed in quadruplicate. Maximum growth rate, lag time and OD max for each strain were calculated using GrowthRates software with default parameters [49].

*Fermentation in beer wort and HPLC analysis*

Fermentations were conducted using a 12°P high-gravity wort at 12°C in 50 mL (micro-fermentations). The 12 °P wort was prepared from a Munton's Connoisseurs Pilsner Lager kit (Muntons plc, England). The worts were oxygenated to 15 mg/L prior to pitching. For the micro-fermentations, the strains were initially grown with constant agitation in 5 mL of wort for 48 hours at 15°C. Following this, 50 mL of fresh wort were inoculated to a final concentration of 15 × 10^6^ viable cells/mL and fermentations were maintained for seven days. Fermentations were weighed every day to calculate the CO_2_ output. The fermentations were maintained until no-CO_2_ lost was observed. At the end of the fermentation, the fermented worts were centrifuged at 9,000xg for 10 min and the supernatant was collected. From this, the concentration of extracellular metabolites was determined using HPLC. Specifically, 20 μL of filtered wort were injected in a Shimadzu Prominence HPLC (Shimadzu, USA) with a Bio-Rad HPX –87H column (Nissen et al., 1997). In this way, the concentrations of glucose, fructose, maltose, maltotriose, ethanol, and glycerol were estimated.

*Data Analysis*

Multiple comparisons across localities were performed utilising a non-parametric Kruskal-Wallis test and Dunn's Multiple Test Comparison. Genomewide *F*is and *F*st data across lineages was compared using paired Student t-test. Spearman rank correlation test and Pearson test were performed to determine correlations between variables. Finally, all analyses were performed utilising GraphPad Prism Software 5.2, except for correlation analysis which were performed in R [50]. In all cases *p*-values < 0.05 were considered as significant.

**References**

1. Sampaio JP, Goncalves P. Natural populations of Saccharomyces kudriavzevii in Portugal are associated with oak bark and are sympatric with S. cerevisiae and S. paradoxus. Appl Environ Microbiol. 2008;74(7):2144-52. doi: 10.1128/AEM.02396-07. PubMed PMID: 18281431; PubMed Central PMCID: PMCPMC2292605.

2. J White T, Bruns T, Lee S, Taylor J, A Innis M, H Gelfand D, et al. Amplification and Direct Sequencing of Fungal Ribosomal RNA Genes for Phylogenetics. 311990. p. 315-22.

3. Pham T, Wimalasena T, Box WG, Koivuranta K, Storgards E, Smart KA, et al. Evaluation of ITS PCR and RFLP for Differentiation and Identification of Brewing Yeast and Brewery 'Wild' Yeast Contaminants. J I Brewing. 2011;117(4):556-68. doi: DOI 10.1002/j.2050-0416.2011.tb00504.x. PubMed PMID: WOS:000303363900010.

4. Peris D, Sylvester K, Libkind D, Goncalves P, Sampaio JP, Alexander WG, et al. Population structure and reticulate evolution of Saccharomyces eubayanus and its lager-brewing hybrids. Mol Ecol. 2014;23(8):2031-45. doi: 10.1111/mec.12702. PubMed PMID: 24612382.

5. Hahne F, LeMeur N, Brinkman RR, Ellis B, Haaland P, Sarkar D, et al. flowCore: a Bioconductor package for high throughput flow cytometry. BMC Bioinformatics. 2009;10:106. doi: 10.1186/1471-2105-10-106. PubMed PMID: 19358741; PubMed Central PMCID: PMCPMC2684747.

6. Sarkar D, Le Meur N, Gentleman R. Using flowViz to visualize flow cytometry data. Bioinformatics. 2008;24(6):878-9. Epub 2008/02/05. doi: 10.1093/bioinformatics/btn021. PubMed PMID: 18245128; PubMed Central PMCID: PMCPMC2768483.

7. Andrews S. FastQC A Quality Control tool for High Throughput Sequence Data2014.

8. Brickwedde A, Brouwers N, van den Broek M, Gallego Murillo JS, Fraiture JL, Pronk JT, et al. Structural, Physiological and Regulatory Analysis of Maltose Transporter Genes in Saccharomyces eubayanus CBS 12357(T). Front Microbiol. 2018;9:1786. doi: 10.3389/fmicb.2018.01786. PubMed PMID: 30147677; PubMed Central PMCID: PMCPMC6097016.

9. Chen S, Zhou Y, Chen Y, Gu J. fastp: an ultra-fast all-in-one FASTQ preprocessor. Bioinformatics. 2018;34(17):i884-i90. Epub 2018/11/14. doi: 10.1093/bioinformatics/bty560. PubMed PMID: 30423086; PubMed Central PMCID: PMCPMC6129281.

10. Peris D, Langdon QK, Moriarty RV, Sylvester K, Bontrager M, Charron G, et al. Complex Ancestries of Lager-Brewing Hybrids Were Shaped by Standing Variation in the Wild Yeast Saccharomyces eubayanus. PLoS Genet. 2016;12(7):e1006155. doi: 10.1371/journal.pgen.1006155. PubMed PMID: 27385107; PubMed Central PMCID: PMCPMC4934787.

11. Bing J, Han PJ, Liu WQ, Wang QM, Bai FY. Evidence for a Far East Asian origin of lager beer yeast. Curr Biol. 2014;24(10):R380-1. doi: 10.1016/j.cub.2014.04.031. PubMed PMID: 24845661.

12. Gayevskiy V, Goddard MR. Saccharomyces eubayanus and Saccharomyces arboricola reside in North Island native New Zealand forests. Environ Microbiol. 2016;18(4):1137-47. doi: 10.1111/1462-2920.13107. PubMed PMID: 26522264.

13. Li H. Aligning sequence reads, clone sequences and assembly contigs with BWA-MEM2013.

14. García-Alcalde F, Okonechnikov K, Carbonell J, Cruz LM, Götz S, Tarazona S, et al. Qualimap: evaluating next-generation sequencing alignment data. Bioinformatics. 2012;28(20):2678-9. doi: 10.1093/bioinformatics/bts503.

15. Li H, Handsaker B, Wysoker A, Fennell T, Ruan J, Homer N, et al. The Sequence Alignment/Map format and SAMtools. Bioinformatics. 2009;25(16):2078-9. doi: 10.1093/bioinformatics/btp352. PubMed PMID: 19505943; PubMed Central PMCID: PMCPMC2723002.

16. Almeida P, Goncalves C, Teixeira S, Libkind D, Bontrager M, Masneuf-Pomarede I, et al. A Gondwanan imprint on global diversity and domestication of wine and cider yeast Saccharomyces uvarum. Nat Commun. 2014;5:4044. doi: 10.1038/ncomms5044. PubMed PMID: 24887054.

17. Scannell DR, Zill OA, Rokas A, Payen C, Dunham MJ, Eisen MB, et al. The Awesome Power of Yeast Evolutionary Genetics: New Genome Sequences and Strain Resources for the Saccharomyces sensu stricto Genus. G3 (Bethesda). 2011;1(1):11-25. doi: 10.1534/g3.111.000273. PubMed PMID: 22384314; PubMed Central PMCID: PMCPMC3276118.

18. DePristo MA, Banks E, Poplin R, Garimella KV, Maguire JR, Hartl C, et al. A framework for variation discovery and genotyping using next-generation DNA sequencing data. Nat Genet. 2011;43(5):491-8. Epub 2011/04/12. doi: 10.1038/ng.806. PubMed PMID: 21478889; PubMed Central PMCID: PMCPMC3083463.

19. Van der Auwera GA, Carneiro MO, Hartl C, Poplin R, Del Angel G, Levy-Moonshine A, et al. From FastQ data to high confidence variant calls: the Genome Analysis Toolkit best practices pipeline. Current protocols in bioinformatics. 2013;43:11.0.1-33. Epub 2014/11/29. doi: 10.1002/0471250953.bi1110s43. PubMed PMID: 25431634; PubMed Central PMCID: PMCPMC4243306.

20. Cingolani P, Platts A, Wang le L, Coon M, Nguyen T, Wang L, et al. A program for annotating and predicting the effects of single nucleotide polymorphisms, SnpEff: SNPs in the genome of Drosophila melanogaster strain w1118; iso-2; iso-3. Fly. 2012;6(2):80-92. Epub 2012/06/26. doi: 10.4161/fly.19695. PubMed PMID: 22728672; PubMed Central PMCID: PMCPMC3679285.

21. Nguyen LT, Schmidt HA, von Haeseler A, Minh BQ. IQ-TREE: a fast and effective stochastic algorithm for estimating maximum-likelihood phylogenies. Mol Biol Evol. 2015;32(1):268-74. Epub 2014/11/06. doi: 10.1093/molbev/msu300. PubMed PMID: 25371430; PubMed Central PMCID: PMCPMC4271533.

22. Hoang DT, Chernomor O, von Haeseler A, Minh BQ, Vinh LS. UFBoot2: Improving the Ultrafast Bootstrap Approximation. Mol Biol Evol. 2018;35(2):518-22. Epub 2017/10/28. doi: 10.1093/molbev/msx281. PubMed PMID: 29077904; PubMed Central PMCID: PMCPMC5850222.

23. Danecek P, Auton A, Abecasis G, Albers CA, Banks E, DePristo MA, et al. The variant call format and VCFtools. Bioinformatics. 2011;27(15):2156-8. Epub 2011/06/10. doi: 10.1093/bioinformatics/btr330. PubMed PMID: 21653522; PubMed Central PMCID: PMCPMC3137218.

24. Li YL, Liu JX. StructureSelector: A web-based software to select and visualize the optimal number of clusters using multiple methods. Mol Ecol Resour. 2018;18(1):176-7. Epub 2017/09/19. doi: 10.1111/1755-0998.12719. PubMed PMID: 28921901.

25. Evanno G, Regnaut S, Goudet J. Detecting the number of clusters of individuals using the software STRUCTURE: a simulation study. Mol Ecol. 2005;14(8):2611-20. Epub 2005/06/23. doi: 10.1111/j.1365-294X.2005.02553.x. PubMed PMID: 15969739.

26. Kopelman NM, Mayzel J, Jakobsson M, Rosenberg NA, Mayrose I. Clumpak: a program for identifying clustering modes and packaging population structure inferences across K. Mol Ecol Resour. 2015;15(5):1179-91. Epub 2015/02/17. doi: 10.1111/1755-0998.12387. PubMed PMID: 25684545; PubMed Central PMCID: PMCPMC4534335.

27. Ramasamy RK, Ramasamy S, Bindroo BB, Naik VG. STRUCTURE PLOT: a program for drawing elegant STRUCTURE bar plots in user friendly interface. SpringerPlus. 2014;3(1):431. doi: 10.1186/2193-1801-3-431.

28. Patterson N, Price AL, Reich D. Population structure and eigenanalysis. PLoS genetics. 2006;2(12):e190-e. doi: 10.1371/journal.pgen.0020190. PubMed PMID: 17194218.

29. Lawson DJ, Hellenthal G, Myers S, Falush D. Inference of Population Structure using Dense Haplotype Data. PLOS Genetics. 2012;8(1):e1002453. doi: 10.1371/journal.pgen.1002453.

30. Browning SR, Browning BL. Rapid and Accurate Haplotype Phasing and Missing-Data Inference for Whole-Genome Association Studies By Use of Localized Haplotype Clustering. The American Journal of Human Genetics. 2007;81(5):1084-97. doi: 10.1086/521987.

31. Cubillos FA, Billi E, Zorgo E, Parts L, Fargier P, Omholt S, et al. Assessing the complex architecture of polygenic traits in diverged yeast populations. Mol Ecol. 2011. Epub 2011/01/26. doi: 10.1111/j.1365-294X.2011.05005.x. PubMed PMID: 21261765.

32. Pickrell JK, Pritchard JK. Inference of Population Splits and Mixtures from Genome-Wide Allele Frequency Data. PLOS Genetics. 2012;8(11):e1002967. doi: 10.1371/journal.pgen.1002967.

33. Patterson N, Moorjani P, Luo Y, Mallick S, Rohland N, Zhan Y, et al. Ancient Admixture in Human History. Genetics. 2012;192(3):1065-93. doi: 10.1534/genetics.112.145037.

34. Milanesi M, Capomaccio S, Vajana E, Bomba L, Garcia JF, Ajmone-Marsan P, et al. BITE: an R package for biodiversity analyses. bioRxiv. 2017:181610. doi: 10.1101/181610.

35. Petr M, Vernot B, Kelso J. admixr—R package for reproducible analyses using ADMIXTOOLS. Bioinformatics. 2019;35(17):3194-5. doi: 10.1093/bioinformatics/btz030.

36. Leppälä K, Nielsen SV, Mailund T. admixturegraph: an R package for admixture graph manipulation and fitting. Bioinformatics. 2017;33(11):1738-40. doi: 10.1093/bioinformatics/btx048.

37. Jombart T. adegenet: a R package for the multivariate analysis of genetic markers. Bioinformatics. 2008;24(11):1403-5. Epub 2008/04/10. doi: 10.1093/bioinformatics/btn129. PubMed PMID: 18397895.

38. Pfeifer B, Wittelsburger U, Ramos-Onsins SE, Lercher MJ. PopGenome: an efficient Swiss army knife for population genomic analyses in R. Mol Biol Evol. 2014;31(7):1929-36. doi: 10.1093/molbev/msu136. PubMed PMID: 24739305; PubMed Central PMCID: PMCPMC4069620.

39. Hellenthal G, Busby GBJ, Band G, Wilson JF, Capelli C, Falush D, et al. A Genetic Atlas of Human Admixture History. Science. 2014;343(6172):747-51. doi: 10.1126/science.1243518.

40. Weir BS, Cockerham CC. Estimating F-Statistics for the Analysis of Population Structure. Evolution. 1984;38(6):1358-70. doi: 10.1111/j.1558-5646.1984.tb05657.x. PubMed PMID: 28563791.

41. Pembleton LW, Cogan NO, Forster JW. StAMPP: an R package for calculation of genetic differentiation and structure of mixed-ploidy level populations. Mol Ecol Resour. 2013;13(5):946-52. Epub 2013/06/07. doi: 10.1111/1755-0998.12129. PubMed PMID: 23738873.

42. Hill WG, Weir BS. Variances and covariances of squared linkage disequilibria in finite populations. Theor Popul Biol. 1988;33(1):54-78. Epub 1988/02/01. PubMed PMID: 3376052.

43. Goudet J. hierfstat, a package for r to compute and test hierarchical F-statistics. Molecular Ecology Notes. 2005;5(1):184-6. doi: 10.1111/j.1471-8286.2004.00828.x.

44. Dray S, Dufour A-B. The ade4 Package: Implementing the Duality Diagram for Ecologists. 2007. 2007;22(4):20. Epub 2007-09-02. doi: 10.18637/jss.v022.i04.

45. Peter J, De Chiara M, Friedrich A, Yue JX, Pflieger D, Bergstrom A, et al. Genome evolution across 1,011 Saccharomyces cerevisiae isolates. Nature. 2018;556(7701):339-44. doi: 10.1038/s41586-018-0030-5. PubMed PMID: 29643504.

46. Yue JX, Liti G. Long-read sequencing data analysis for yeasts. Nat Protoc. 2018;13(6):1213-31. Epub 2018/05/05. doi: 10.1038/nprot.2018.025. PubMed PMID: 29725120.

47. Bateman A, Coin L, Durbin R, Finn RD, Hollich V, Griffiths-Jones S, et al. The Pfam protein families database. Nucleic acids research. 2004;32(Database issue):D138-D41. doi: 10.1093/nar/gkh121. PubMed PMID: 14681378.

48. Kessi-Perez EI, Araos S, Garcia V, Salinas F, Abarca V, Larrondo LF, et al. RIM15 antagonistic pleiotropy is responsible for differences in fermentation and stress response kinetics in budding yeast. FEMS Yeast Res. 2016. doi: 10.1093/femsyr/fow021. PubMed PMID: 26945894.

49. Hall BG, Acar H, Nandipati A, Barlow M. Growth rates made easy. Mol Biol Evol. 2014;31(1):232-8. doi: 10.1093/molbev/mst187. PubMed PMID: 24170494.

50. Development Core Team R. R Core Team. R A Language and Environment for Statistical Computing 2014. 2008.
