# Supplementary material for: An Out-of-Patagonia migration explains the worldwide diversity and distribution of Saccharomyces eubayanus lineages
Source: PLoS Genet. 2020 May 1;16(5):e1008777. doi: 10.1371/journal.pgen.1008777 (PMC7219788; doi:10.1371/journal.pgen.1008777)
Supplement: S2 Text — (DOCX) [file pgen.1008777.s008.docx]

**Supplementary Note**

**Detecting evidence of gene flow among *S. eubayanus* populations using admixture graphs**

Genotype frequencies in linked populations are correlated. In a case where three related populations, in which one separated earlier from a common ancestor and the other two later in time, the two closest populations will, under certain assumptions, have more correlated genotype frequencies than they will have with the population that drifted first. Moreover, their genotype frequencies should be equally drifted to the earliest divergent population. However, if a single (or short-lasting) event of gene flow occurred between the first divergent population and one of the other two, the genotype frequency drift between the two admixed populations would be different from that expected under the assumption of no gene flow. These events of gene flow in a set of populations can be described using admixture graphs [1]. The f2, f3, and f4 test are widely used to obtain the correlations in genotype frequencies between populations. The admixturegraph R-package fit the values of f2, f3, and f4 obtained from a set of populations and evaluates the fit of these statistics to a user-imputed phylogeny model. In Fig 5D we show the fit of f4 statistics (four population tests) to the model where no gene flow occurred between the HOL, PA, PB-1, PB-2, and PB-3 populations after their respective splits. Error bars show the calculated f4 values for these populations (Z-scores). At the same time, the circles indicate the f4 value expected under the model of no-gene flow (Fig 5C), which are colored green for those that fall within the calculated f4, and red for those that do not fit. Different admixture models were tested (Supplemental Fig 4E) for their fit to the calculated f4 statistics, and the model that best fits the data is shown in Fig 5EF.

1. Patterson N, Moorjani P, Luo Y, Mallick S, Rohland N, Zhan Y, et al. Ancient Admixture in Human History. Genetics. 2012;192(3):1065-93. doi: 10.1534/genetics.112.145037.
